# Ultrasound with microbubbles improves memory, ameliorates pathology and modulates hippocampal proteomic changes in a triple transgenic mouse model of Alzheimer's disease

**DOI:** 10.7150/thno.44152

**Published:** 2020-09-26

**Authors:** Yuanyuan Shen, Lingchen Hua, Chih-Kuang Yeh, Liming Shen, Ming Ying, Zaijun Zhang, Gongping Liu, Shupeng Li, Siping Chen, Xin Chen, Xifei Yang

**Affiliations:** 1 National-Regional Key Technology Engineering Laboratory for Medical Ultrasound, School of Biomedical Engineering, Health Science Center, Shenzhen University, Shenzhen, 518060, China.; 2 Key Laboratory of Modern Toxicology of Shenzhen, Shenzhen Medical Key Discipline of Health Toxicology (2020-2024), Shenzhen Center for Disease Control and Prevention, Shenzhen, 518055, China.; 3 Department of Biomedical Engineering and Environmental Sciences, National Tsing Hua University, Hsinchu, 30013 Taiwan.; 4 College of Life Sciences and Oceanography, Shenzhen University, Shenzhen, 518060, China.; 5 Institute of New Drug Research and Guangzhou Key Laboratory of Innovative Chemical Drug Research in Cardio-cerebrovascular Diseases, College of Pharmacy, Jinan University Guangzhou, 510632, China.; 6 Department of Pathophysiology, School of Basic Medicine and the Collaborative Innovation Center for Brain Science, Key Laboratory of Ministry of Education of China for Neurological Disorders, Tongji Medical College, Huazhong University of Science and Technology, Wuhan, 430030, China.; 7 State Key Laboratory of Oncogenomics, School of Chemical Biology and Biotechnology, Peking University Shenzhen Graduate School, Shenzhen 518055, China.

**Keywords:** Triple transgenic mouse model of Alzheimer's disease, blood-brain barrier, focused ultrasound, microbubbles, behavioral tests

## Abstract

Alzheimer's disease (AD) is a progressive neurodegenerative disease manifested by cognitive impairment. As a unique approach to open the blood-brain barrier (BBB) noninvasively and temporarily, a growing number of studies showed that low-intensity focused ultrasound in combination with microbubbles (FUS/MB), in the absence of therapeutic agents, is capable of ameliorating amyloid or tau pathology, concurrent with improving memory deficits of AD animal models. However, the effects of FUS/MB on both the two pathologies simultaneously, as well as the memory behaviors, have not been reported so far.

**Methods:** In this study, female triple transgenic AD (3×Tg-AD) mice at eight months of age with both amyloid-β (Aβ) deposits and tau phosphorylation were treated by repeated FUS/MB in the unilateral hippocampus twice per week for six weeks. The memory behaviors were investigated by the Y maze, the Morris water maze and the step-down passive avoidance test following repeated FUS/MB treatments. Afterwards, the involvement of Aβ and tau pathology were assessed by immunohistochemical analysis. Neuronal health and phagocytosis of Aβ deposits by microglia in the hippocampus were examined by confocal microscopy. Further, hippocampal proteomic alterations were analyzed by employing two-dimensional fluorescence difference gel electrophoresis (2D-DIGE) combined with mass spectrometry.

**Results:** The three independent memory tasks were indicative of evident learning and memory impairments in eight-month-old 3×Tg-AD mice, which developed intraneuronal Aβ, extracellular diffuse Aβ deposits and phosphorylated tau in the hippocampus and amygdala. Following repeated FUS/MB treatments, significant improvement in learning and memory ability of the 3×Tg-AD mice was achieved. Amelioration in both Aβ deposits and phosphorylated tau in the sonicated hemisphere was induced in FUS/MB-treated 3×Tg-AD mice. Albeit without increase in neuron density, enhancement in axonal neurofilaments emerged from the FUS/MB treatment. Confocal microscopy revealed activated microglia engulfing Aβ deposits in the FUS/MB-treated hippocampus. Further, proteomic analysis revealed 20 differentially expressed proteins, associated with glycolysis, neuron projection, mitochondrial pathways, metabolic process and ubiquitin binding etc., in the hippocampus between FUS/MB-treated and sham-treated 3×Tg-AD mice.

**Conclusions:** Our findings reinforce the positive therapeutic effects on AD models with both Aβ and tau pathology induced by FUS/MB-mediated BBB opening, further supporting the potential of this treatment regime for clinical applications.

## Introduction

Alzheimer's disease (AD) is the most common age-related neurodegenerative disorder, and manifests as progressive cognitive impairment (i.e. memory loss) over decades. The prevalence of AD in patients is expected to grow exponentially over the next few decades, presenting an ever-increasing economic and care burden 1. Unfortunately, medications currently available for AD may only temporarily and modestly improve the cognitive symptoms. Thus, great efforts have been made to explore viable treatment strategies for AD.

The two major pathological hallmarks of AD are extracellular amyloid-β (Aβ) peptide deposition into plaques, and neurofibrillary tangles composed of hyperphosphorylated tau proteins, which lead to irreversible death of neurons in the cerebral cortex and hippocampus 2, 3. Thus, the development of therapeutic agents aimed at clearing Aβ plaques production or inhibiting tauopathy have been the most popular strategy 4. However, the therapeutic efficiency of these therapeutic agents is severely limited due to the innate hindrance from the blood-brain barrier (BBB), which plays a critical role in maintaining a highly regulated central nervous system milieu by preventing the entry of most molecules from the blood into the brain 5. Low-intensity focused ultrasound combined with circulating microbubbles (FUS/MB) has been demonstrated to be capable of opening the BBB in cumulative preclinical studies 6-8. FUS/MB's potential in enhancing the delivery of antibodies to the brain has also been proven in mouse models of AD 9-11. Enhanced delivery of anti-amyloid-beta peptide (anti-Aβ) antibodies was found in the FUS/MB-targeted brain regions of two AD mice models (B6C3-Tg and PDAPP, center frequency: 0.69 MHz, peak rarefactional pressure: 0.67-0.8 MPa) 9. Even a single application of magnetic resonance-guided focused ultrasound in the presence of microbubbles (MBs) (MRgFUS/MB, 0.558 MHz, 0.3 MPa for 120 s, 10-ms pulse length at 1 Hz) to four right-hemisphere spots in the TgCRND8 mouse model of AD resulted in successful delivery of anti-Aβ antibody BAM-10 and effectively reduced plaque load four days later 10; however, the memory-related behavioral test was not incorporated in this study. Using a different mouse model of AD involving P301L human tau transgenic pR5 mouse, the efficacy of an anti-tau antibody delivered by a scanning ultrasound combined with MBs (SUS/MB, sonication to the entire forebrain, 1 MHz, 0.7 MPa, 10% duty cycle at 1 Hz) was investigated 11. The fluorescence intensity of the labeled antibodies in the treated mouse brains increased by eleven-fold compared with the brains of untreated mice. The anxiety-like behavior, which was assessed by elevated plus maze, was also greatly improved. Particularly, the approach is showing great potential towards clinical application, given a pilot clinical trial successfully demonstrating the safety of repeated FUS/MB in opening the BBB of five AD patients 12.

Aside from being a potential method to enhance the delivery of exogenous antibodies, a growing number of studies found that FUS/MB alone could also result in beneficial effects in a range of transgenic AD animal models, even without administration of any therapeutics 13-16. BBB compromise was demonstrated by capillary leakages of blood-derived macromolecules or exogenous tracers, with degeneration of brain capillary pericytes and endothelial cells in transgenic AD models 17-24. Despite this compromise, these studies suggested that transient BBB opening by FUS/MB still plays a positive role 13-16. A significant decrease in plaque burden was observed in TgCRND8 mice given only a single FUS/MB treatment (0.558 MHz, 0.3 MPa for 120 s, 10-ms pulse length at 1 Hz), which was revealed by reductions of the mean plaque size (20%) and total Aβ surface area (13%) 15. An ensuing study utilizing a Y-maze test showed remarkable improvement in the spatial memory of AD mice treated weekly with MRgFUS/MB (1.68 MHz, maximum peak pressure: 1.18 MPa for 120 s, 10 ms pulse length at 1 Hz) in the bilateral hippocampus for one month, along with decreased amyloid burden and increased neuronal plasticity 14. In addition, APP23 mice treated by repeated SUS/MB (1 MHz, 0.7 MPa, 10% duty cycle at 1 Hz) over 6-7 weeks on the entire brain displayed improved performance on three memory tasks: the Y-maze, the novel object recognition test and the active place avoidance task 16. The phenomenon of increased plaque phagocytosis in FUS/MB-treated mouse brains by microglia was revealed; this phenomenon was likely activated by the facilitated entry of endogenous immunoglobulin in the blood stream into the brain, which was concurrent with the opening of BBB 15, 16. Of note, proximate studies commenced investigating the effect of the ultrasound on tau pathology in detail, without any antibody intervention 25, 26. One study found that the immune response was well correlated with the amelioration of tau pathology in the hippocampus of rTg4510 mice after unilateral FUS/MB-facilitated BBB opening (1.5 MHz, 0.62 MPa for 120 s, 6.7-ms pulse length at 10 Hz) 25. The other study also supported the potential of this technique to clear intraneuronal phosphorylated tau with a possible autophagy mechanism 26.

Given the promising results of the above studies, the present study investigated the effects of FUS/MB treatment in a triple transgenic AD (3×Tg-AD) mouse model, in which mice develop both Aβ and tau pathology predominantly in the hippocampus, amygdala, and the cerebral cortex 27, 28. The memory and cognitive performance of the mice after treatment was assessed by three independent memory-related behavioral tests: Y maze, Morris water maze and step-down passive avoidance test. The involvement of Aβ and tau pathology, as well as neuronal health and phagocytosis of Aβ deposits by microglia in the hippocampus, were also studied by immunohistological analysis. To provide new clues for understanding the effects induced by FUS/MB treatment in this AD model, hippocampal proteomic alterations were analyzed by employing two-dimensional fluorescence difference in-gel electrophoresis (2D-DIGE) combined with mass spectrometry.

## Materials and Methods

### Animal models

Eight-month-old female 3×Tg-AD mice harboring the human mutations of APP_swe_, PS1_M126V_, and Tau_P301L_ (strain: B6; 129-Psen1^tm1Mpm^ Tg [APPSwe, tauP301L] 1Lfa/Mmjax), as well as wild type (WT) mice (strain: B6129SF2/J) with the same genetic background, were used in this study (Jackson Laboratory, Maine, USA). This study used eight-month-old 3×Tg-AD mice because they exhibit both Aβ depositon and tau hyperphosphorylation in the hippocampus and cortex 27, 28. The mice were maintained under standard laboratory conditions (relatively constant temperature: 23°C-25°C, humidity: 55% ± 5%, 12 h light/dark cycle) with food and water available *ad libitum*. Animal care and experiments were approved by the Animal Care and Use from the Committee of the Experimental Animal Center at Shenzhen University (Approval No.: AEWC 20160110).

### Preparation and characterization of MBs

MBs with a lipid shell and perfluoropropane gas core were prepared as described previously 29, 30. The molar ratio of 1,2-distearoyl-sn-glycero-3-phosphocholine (DSPC) and N-(carbonyl-methoxypolyethylene glycol-2000)-1,2-distearoyl-sn-glycero-3-phosphoethanolamine (DSPE-PEG2000) (Lipoid, Ludwigshafen, Germany) was 9:1. After preparation, the concentration and particle size distribution of the prepared MBs were measured by a Coulter Counter Multisizer IV (Beckman Coulter Inc., Miami, FL, USA) after dilution with Isoton II. The structure of MBs was visualized by a microscope (BX-53, Olympus Corporation, Tokyo, Japan). Freshly prepared MBs were polydispersed with a concentration ranging from 1-3×10^9^/mL. To investigate the lifespan of MBs in the brain, contrast-enhanced ultrasound imaging was performed (n = 3) using a small animal acoustic imaging system (Vevo^®^ LAZR; VisualSonics Inc., Toronto, Canada) with a 40-MHz transducer. To eliminate the effect of the skull, craniotomy surgery was performed on mice (approximately 0.5 × 0.5 cm^2^). Serial images were acquired before and after the injection of MBs. Through the time intensity curves of the images, the lifespan of MBs could be determined.

### Sonication apparatus and procedure

**Figure 1:**
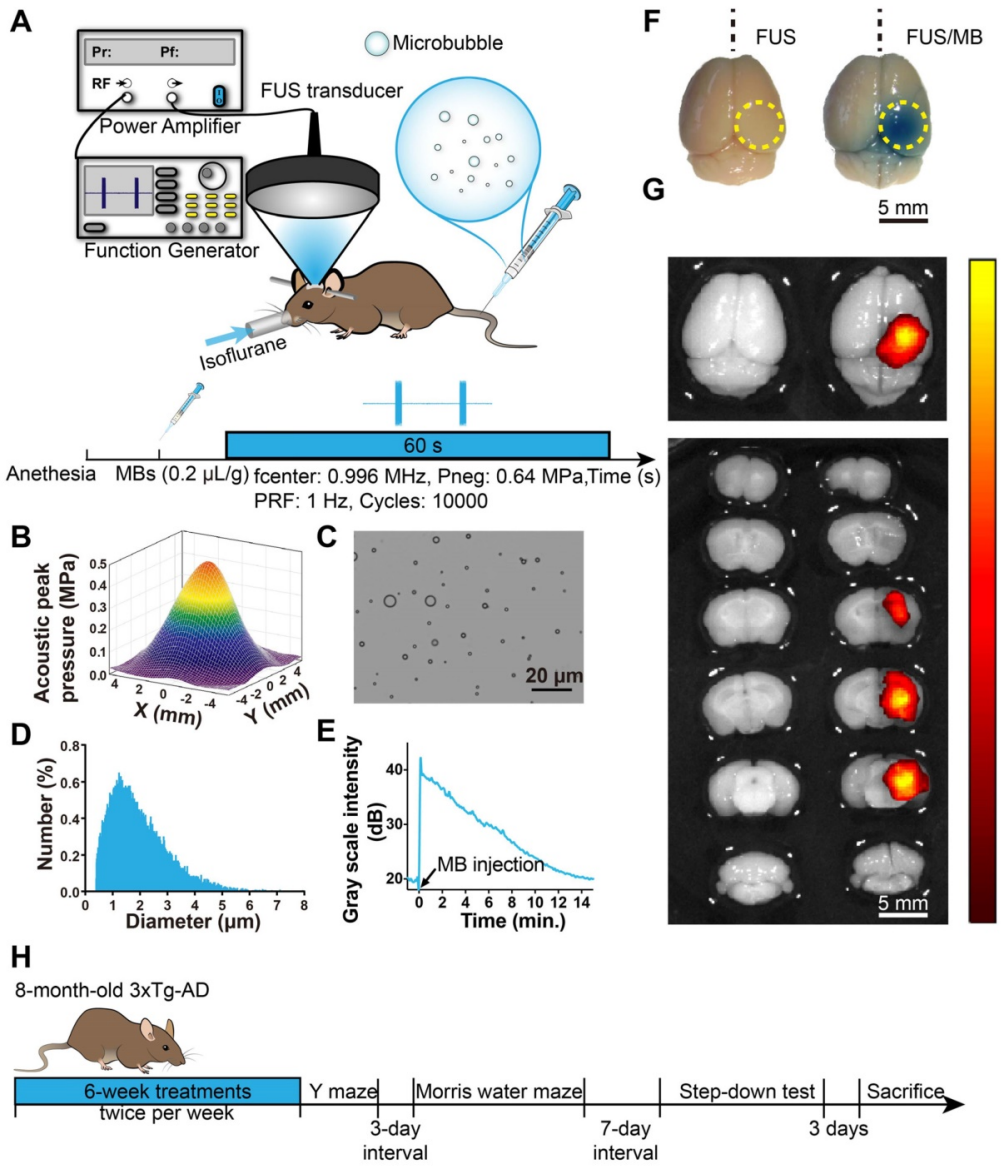
** BBB opening induced by FUS/MB treatment.** (**A**) Illustration of the experimental setup and the timeline of the sonication procedure. (**B**) The acoustic peak pressure profile of the focused ultrasound beam at its lateral focal plane (the lateral width at half maximum intensity: 5.0 mm). (**C**) Representative photomicrograph of MBs with a lipid shell and perfluoropropane core. Scale bar: 20 µm. (**D**) MB size distribution. MBs were polydispersed with diameters ranging from 0.4 µm to 8 µm. (**E**) The time-intensity curve of contrast-enhanced B-mode imaging of the brain after MB injection. The signal intensity gradually dropped to the half level about 7 min later and to the initial amplitude about 15 min later. (**F**) BBB opening area after a one-time FUS/MB treatment revealed by Evans blue (EB) extravasation (yellow dotted circle). Mice were unilaterally sonicated on the right hemisphere and the left hemisphere was used as the control. Left: treated by FUS only without MB injection. Right: treated by FUS/MB. The blue coloration could be visible only in the FUS/MB-treated site. The center of the target region was positioned 1.5-mm anterior to the lambda and 2.0-mm laterally towards the right hemisphere. Scale bar: 5 mm. (**G**) Fluorescence images of EB extravasated into the brain interstitium of the same brains in (F). Scale bar: 5 mm. (**H**) Scheme of treatment and behavioral tests in eight-month-old 3×Tg-AD mice receiving sham or FUS or FUS/MB treatment twice per week for a total duration of six weeks.

The experimental setup is illustrated in Figure 1A. A focused ultrasound beam was generated by an in-house-manufactured single-element spherical transducer (Center frequency: 0.996 MHz, focal length: 80 mm), which was immersed in a cone filled with degassed water. The tip of the cone was capped with a thin polyurethane membrane providing an acoustic window that allows an ultrasound beam to pass through. The transducer with the cone was mounted on a 3-D positioning system (RWD Life Science, Shenzhen, China) and driven by a 50-dB power amplifier (2100L, Electronics & Innovation, Rochester, NY, USA). The excitation waveform was generated by a function generator (AFG3102C, Tektronix, Beaverton, OR, USA).

The pressure amplitudes and beam dimensions of the transducer mounted with the cone were measured using a needle hydrophone (HGL-0200, Onda Corporation, Sunnyvale, CA, USA) in an Acoustic Intensity Measurement System (Onda Corporation, Sunnyvale, CA, USA). The focal point of the ultrasound beam was 2.5 mm beneath the center of the cone tip. Figure 1B showed the pressure field in the lateral plane. The lateral and axial full-widths of the beam at half maximum intensity were 5.0 mm and 55.0 mm, respectively.

Animals were placed in the prone position and anesthetized with 1.5% isoflurane (RWD Life Science, China), with their heads immobilized by a stereotaxic apparatus (RWD Life Science, Shenzhen, China) and depilated to minimize acoustic impedance mismatch. The body temperature of each mouse was maintained through a heating pad. Ultrasound gel was applied between the polyurethane membrane and mouse scalp skin as a coupling medium to allow ultrasonic waves to transmit to the mouse brain with minimal attenuation. Using the 3-D positioning system of the stereotaxic apparatus with 0.01mm precision, a metal pointer was used to locate the lambda on the skull sutures and then replaced with the FUS transducer, the focus of which was aligned with lambda. The center of the target region was positioned 1.5-mm anterior to the lambda and 2.0-mm laterally towards the right hemisphere to ensure the ultrasound beam covering the hippocampus formation. The sonication procedure is illustrated in Figure 1A. A bolus of diluted MBs (0.1 mL) was injected through the tail vein of the mouse at a dose of 0.2 µL/g of body weight. Next, pulsed FUS was applied on the right hemisphere for 60 seconds with a peak-rarefactional pressure amplitude of 0.64 MPa, a burst length of 10 ms and a repetition frequency of 1 Hz. During the experiment, the intraveneous injection was performed by a skilled technician to reduce stress as much as possible.

### FUS/MB treatment

To assess the opening of the BBB, Evans blue dye (EB), which binds to albumin (molecular weight: 67 kDa) after entering the circulation, was injected intravenously. Two hours later, the animals were sacrificed and the brains were extracted for fluorescence imaging and histological examination. In order to ensure that our targeting method was reproducible and that the FUS beam consistently covered the hippocampus of the murine brain, a separate set of experiments was first performed on WT mice, which either received a one-time FUS treatment without MB (FUS, n = 10) or FUS treatment combined with MB (FUS/MB, n = 10). After FUS/MB sonication, EB extravasation showed that the targeting was within 0.5 mm of the intended focus and the diameter of the BBB opening area in the lateral direction was 4.6 ± 0.4 mm, which corresponded to the lateral FUS beam focus dimension. To examine the time course of the BBB, another cohort of transgenic mice underwent FUS/MB treatment, and EB was injected at the time points of 0 h, 1.5 h, 3 h, 6 h and 24 h after the treatment (n = 3-5 for each group), circulating for another two hours. The brains were extracted on ice after cardiac perfusion for immunofluorescence analysis.

The age-matched 3×Tg-AD female mice were randomly divided into three groups. The sham group received MB injections but without FUS sonication (sham, n = 10). Another group received FUS treatment alone without MB injections (FUS, n = 10). The mice in the third group were administered FUS treatment combined with MB injections (FUS/MB, n = 10). Each treatment regimen was performed twice a week over a six-week period. The age-matched WT mice received no treatment (WT, n = 10). The body weights of the animals were monitored.

### Behavioral tests

After all the treatments, WT mice and 3×Tg-AD female mice in the sham, FUS and FUS/MB groups were examined by Y-maze, Morris water maze and step-down test, sequentially, with a three-day and a seven-day interval respectively (Figure 1H). All behavioral tests were conducted in a quiet room with infrared illumination and the mice were kept in the room for at least 1 h before testing.

### Y-maze test

The Y-maze apparatus consisted of three identical arms made of black plastic, each 44 cm long and 15 cm wide, with walls 10 cm high, allowing the mice to see distal spatial landmarks (Figure 3A). This test relies upon the innate tendency of the mice to explore novel environments. Three arms were randomly designated as the start arm, novel arm, and other arm. In the training trial, the novel arm was blocked off. The mice were placed into the start arm with their heads pointing away from the center of the maze and allowed to explore the other arm for 10 min. After a 1 h interval, the mice were returned to explore the maze freely for 5 min, with all three arms open (test trial). All the trials were automatically recorded by a video camera. The number of entries into, the time spent and the distance travelled in each arm were determined from the video recording. The percentages of these three parameters over the total ones were calculated. Total traveling distance and average speed were also recorded.

### Morris water maze test

WT mice and 3×Tg-AD mice in the sham, FUS and FUS/MB groups were trained in an open circular pool (diameter: 170 cm) filled with water at a depth of 30 cm and maintained at 22 °C ± 1°C as described previously 31, 32. For data collection, the maze was divided into four equal quadrants (I-IV) by designating two orthogonal axes, the end of which demarcated four cardinal points: north (N), south (S), east (E), and west (W), as illustrated in Figure 4A. The trace of the mouse was recorded by a video camera (SSC-DC488P, SONY, Japan) hung above the center of the pool. The spatial acquisition task was conducted in four trials per day with a 15 s inter-trial interval, over five consecutive days. The escape platform (10 cm in diameter) was positioned in the middle of quadrant II (target quadrant), approximately 1 cm below the surface of the water. The mouse was gently released into the water from different starting points and allowed to locate the hidden platform within 60 s. If the mouse failed to find the platform within 60 s, it was manually guided to the platform. A probe trial was administered six days after the last spatial acquisition day to evaluate the long-term memory after the treatments 32, 33. The platform was removed from the target quadrant. The mouse was placed into the water in a novel start position, 180° from the original platform position, and allowed to swim for 120 s. Subsequent data including escape latency in spatial acquisition days, latency to first target-site crossover (probe time), number of platform-site crossovers during the probe trial, percent time in target quadrant during the probe trial, and percent distance travelled in the target quadrant during the probe trial was analyzed by the tracking software of the system.

### Step-down test

The one-trial step-down test was employed to measure inhibitory avoidance and long-term memory, which was composed of a 5 min training session, followed 24 h later by a 5 min test session as illustrated in Figure 5A. A chamber with dimensions around 18 (h) × 12 (w) × 12 (d) cm was used. The floor consisted of an electrified grid of parallel 0.1-cm copper bars spaced 0.5 cm apart and a small elevated rubber platform (2.4 cm diameter) in a corner of the chamber. In the training session, animals were gently placed onto the platform with their noses pointing to the bottom corner. Once stepping down with their four paws on the electrified grid, the mice received an immediate electric shock (36V, AC). Instinctively, they showed a tendency to jump up the platform to avoid the shock. The time it took the mouse to step down from the platform onto the grid (step-down latency) and the number of times stepped down during the training period (error counts) were recorded. In the test session, the same procedures were conducted. After the test session, the apparatus was carefully cleaned to reduce the possibility of odor interference.

### Histology

#### Histological staining

Four mice in each group were used for immunohistochemical or immunofluorescence analysis. Mice were anesthetized and transcardially perfused with saline, followed by 20 mL of 4% paraformaldehyde (PFA). Brains were then extracted on ice and fixed in 4% PFA overnight. To assess the safety of FUS/MB treatment, brains were embedded in paraffin and sliced coronally and serially at 7-μm thickness. Sections were stained with hematoxylin and eosin (H&E) and toluidine blue (Nissl) in order to examine microvascular injury and neuronal damage.

For immunohistochemical and immunofluorescence analysis, animals were sacrificed and transcardially perfused three days after all the behavioral tests. Brains were dissected and immersed in 10%, 18% and 30% sucrose solution at 4 °C for cryoprotection. The brains were then embedded with optimum cutting temperature compound (Sakura Finetek, Zoeterwoude, The Netherlands), cryosectioned serially at a thickness of 10 μm and mounted on adhesion slides (SuperFrost^TM^ Plus, Fisher Scientific, USA). To visualize Aβ and p-tau pathology, Aβ immunoreactivity was probed with 6E10 antibody (1:500, 803016, BioLegend), while tau was probed with AT180 and AT8 antibodies (1:500, MN1040 and MN1020, ThermoFisher), followed by detection with HRP/DAB (ABC) Detection IHC kit (Abcam). To visualize fibrinogen leakage, polyclonal rabbit anti-human fibrinogen (1:500, A0080, Dako) was used, which recognizes both the monomeric form of fibrinogen and fibrinogen-derived fibrin polymers and crossreacts with mouse fibrinogen and fibrin 18. To visualize brain microvessels, sections were incubated with Dylight 594-conjugated *L. esculentum* lectin (1:200, Vetor Laboratories). Other primary antibodies used for detecting neurons (NeuN and SMI312) and microglia (Iba1) were anti-NeuN (1:500, ab177487, Abcam), pan-axonal neurofilament antibody SMI312 (1:500, 837904, BioLegend), and anti-Iba1 (1:500, 019-19741, Wako). The secondary antibodies used were donkey Alexa 568-conjungated anti-rabbit IgG (1:200, A10042, Invitrogen), donkey Alexa 488-conjungated anti-mouse IgG (1:200, A21202, Invitrogen) and donkey Alexa 488-conjungated anti-rabbit IgG (1:400, A21206, Invitrogen). Sections were coverslipped with DAPI (4',6-diamidino-2-phenylindole) fluorescence mounting medium (Dako) to counterstain the nuclei.

#### Image acquisition and quantification

To compare the non-sonicated and sonicated hemispheres of the same brain, bright field images of the whole brain sections from the FUS/MB group that underwent H&E, Nissl staining, as well as Aβ and p-tau staining, were automatically scanned using a 20× objective with an Aperio Versa 8 system (Leica Microsystems). To compare the differences among the four groups, images of subregions of the brain sections were acquired by another microscope (BX53; Olympus Corporation, Japan). Fluorescense images were acquired with a confocal laser scanning microscope (LSM 880, Carl ZEISS, Germany). The large field images covering the hippocampus were obtained using a 10× objective and the images were stitched. To visualize the microglia and Aβ deposits, Z-stacks were acquired using a 63× oil objective with a Z-step size of 0.75 μm.

Image quantification was performed blinded using ImageJ software (NIH, Bethesda, MD, USA). Briefly, images were first converted into 8-bit grey scale. After delineating the region of interest, the threshold tool was applied to adaptively discriminate positive staining from the background. Then, immunopositive area percentage in the cortex, hippocampus, or amygdala was obtained and normalized to the area of the field.

For quantification of extravascular fibrin(ogen), five non-adjacent sections (~500 μm apart) were used in each animal (n = 5). For Aβ and p-tau pathology, brain sections were selected from the medial hippocampus (~-2.46 mm posterior to bregma), with three sections per position (equally ~30 μm apart). Immunopositive areas were obtained from the cortex, cornu ammonis (CA) subregion, or amygdala. For NeuN-positive neurons, stitched confocal images were acquired using a 10× objective and the immunopositive area percentage in the hippocampus was determined. For SMI312-positive neurofilaments, the immunopositive area percentages in the CA1 and CA3 subfields were quantified. For the assessment of microglial morphologies, skeleton analysis was carried out on fluorescence images of brain sections stained by anti-Iba1 antibodies using the protocol described in previous publication 34. To better visualize the phagocytosis of Aβ deposits by microglia, three-dimensional (3D) reconstruction was established by Imaris 8.3 (Bitplane).

### Hippocampal proteomics analysis

#### Protein sample preparation

After all the behavioral tests were completed, six mice in each group were euthanized. Their brains were excised and the hippocampi were isolated on ice then stored at -80 °C. To extract proteins for DIGE analysis, the samples were suspended in DIGE-specific lysis buffer (7 M urea, 2 M thiourea, 30 mM Tris-HCl, 4% CHAPS, pH 8.5) and sonicated for 2 min using a sonic dismembrator (Model 550, Fisher Scientific, USA). The samples were incubated on ice for 30 min, and centrifuged subsequently at 20,000 g at 4 °C for 60 min. The supernatants were ultrafiltered in a centrifugal filter (Merck Millipore Ltd., Billerica, MA, USA) to remove salt and other impurities, and then resuspended in DIGE-specific lysis buffer. The protein solutions were collected and the protein concentrations were determined using a 2-D Quant Kit (GE Healthcare, USA) in accordance with the manufacturer's protocol.

#### DIGE labeling of proteins

All the samples from WT mice, and 3×Tg-AD mice receiving sham, FUS, or FUS/MB treatment were diluted to a concentration of 5 μg/μL with DIGE-specific lysis buffer. The protein sample (25 μg) was labeled with 200 pM Cy3 (GE Healthcare, USA) or Cy5 dye (GE Healthcare, USA). The internal standard prepared by pooling together equal aliquots of all the samples was labeled with Cy2 (GE Healthcare, USA). The CyDye stock with a final concentration of 1 nM/μL was prepared by reconstitution in 99.8% anhydrous N, N-Dimethylformamide (DMF, Sigma 227056). Protein labeling was performed by incubation with the working solution of each CyDye (200 pM/μL) on ice in the dark for 30 min, and terminated by the addition of 10 mM lysine (Sigma, USA) for 10 min at 4 °C in the dark. Dye swap was performed with each of the sample types in the study such that an equal number were labeled with Cy3 as with Cy5. After labeling, the Cy2-, Cy3-, and Cy5-labeled samples were mixed and then resolved in rehydration buffer (7 M urea, 2 M thiourea, 2% CHAPS, 2.8% DTT, 0.5% IPG buffer (pH 3-11 NL) and 0.002% bromophenol blue) to a final volume of 450 μL prior to transfer onto immobilized pH gradient strips.

#### 2D difference gel electrophoresis

The first dimension was performed using an Ettan IPGphor Isoelectric Focusing System (GE Healthcare, USA). The strips were first rehydrated and then isoelectric focusing (IEF) was performed. After IEF, a two-step equilibration procedure was performed as described previously 32. The equilibrated strips were loaded on the top of 12.5% SDS-PAGE gels and covered with 0.5% (w/v) ultra-low melting point agarose sealing solution (25 mM Tris, 192 mM glycine, 0.1% SDS, 0.5% (w/v) agarose, 0.02% bromophenol blue). In the second dimension, protein separation employed an Ettan DALTsix Electrophoresis System (GE Healthcare) with the running buffer (25 mM Tris, 192 mM lycine, 0.1% SDS, pH 8.3) at 12 °C through the following steps: 1 W/gel for 1 h, subsequently 11 W/gel for 5 h in the dark.

#### Gel imaging

Gels were scanned in a Typhoon TRIO Variable Mode Imager (GE Healthcare, USA). They were prescanned using a low-resolution setting (1000 µm resolution) to optimize final imaging settings. The final image was scanned at a 100 μm resolution and the maximum pixel intensity of all gel images was ensured to be within a range of 40,000-60,000 pixels.

#### Image analysis

DIGE gel images were analyzed using the DeCyder software package (Version 6.5, GE Healthcare, USA) with the differential in-gel analysis and the biological analysis modules to analyze protein spots. The volume of each protein spot in the Cy3 or Cy5 channel was normalized by the volume of the corresponding Cy2 spot. These normalized values were compared across the gels among the replicate groups. Differentially expressed protein spots (Student's *t* test, *p* < 0.05) were manually excised from the stained gel and digested as described previously for further identification by mass spectrometry 33.

#### Mass spectrometry

Protein identification was carried out by matrix-assisted laser desorption/ionization time-of-flight tandem mass spectrometry (MALDI-TOF-MS/MS, SCIEX TOF/TOF™ 5800 System, AB SCIEX, Framingham, MA, USA). Peptide extracts (0.6 μL) were crystallized with 10 mg/mL α-cyano-4-hydroxycinnamic acid (CHCA) in 0.1% TFA and 50% acetonitrile (ACN) directly onto the target, and dried at room temperature. The spectra were externally calibrated. Protein searching were conducted against the SwissProt *Mus musculus* database housed in MASCOT (Matrix Science, UK) with a mass measurement tolerance of 100 ppm in MS mode and 0.5 Da in MS/MS mode. Fixed carbamidomethyl modification was taken into account and up to two missed cleavages per peptide were allowed.

### Bioinformatics analysis

Functional annotation of differentially expressed proteins was performed with the Database for Annotation, Visualization and Integrated Discovery Resource (DAVID, https://david.ncifcrf.gov). Gene ontology terms (cut-off p-value: *p* < 0.05) included biological processes, molecular functions, cellular components. The protein-protein interaction (PPI) networks of the identified differentially expressed proteins were analyzed by using a web-based tool OMICBEAN (http://www.omicsbean.com).

### Western blot analysis

To confirm the differentially expressed proteins revealed by 2D-DIGE, the expression of the proteins was further measured by western-blot analysis. Hippocampal proteins from WT mice, as well as 3×Tg-AD mice that received either sham or FUS/MB treatment, were extracted using lysis buffer (Beyotime, China) with protease and phosphatase inhibitor cocktail (Thermo Scientific, USA). Protein samples of two mice were used for each group, and testing was performed in triplicate. The lysate was centrifuged at 10,000 g for 15 minutes at 4°C. Then the supernatant was collected and the protein concentration was estimated using micro BCA protein assay kit (Thermo Scientific, USA). Protein samples were mixed with loading buffer and heated at 100°C for 5 min, then separated on 12% sodium dodecyl sulfate polyacrylamide gel electrophoresis (SDS-PAGE) and transferred onto polyvinylidene fluoride membranes, which were blocked in TBST (150 mM NaCl, 10 mM Tris, 0.1% Tween-20, pH 8.0) containing 5% non-fat milk. The blocked membranes were incubated with anti-β-actin (1:1000, Santa Cruz Biotechnology, sc-47778), anti-SYN1 (1:1000, Abcam, ab18814), anti-PGAM1 (1:1000, Abcam, ab184232) and anti-UHCL1 (1:1000, Santa Cruz Biotechnology, sc-271639) in TBST buffer overnight at 4°C. After washing in TBST, the membranes were incubated with a 1:3000 dilution of anti-mouse or anti-rabbit IgG HRP secondary antibody (Stressgen, U.S.A.). After that, they were diluted in TBST for 1 h and then developed using chemiluminescence reagents from an ECL kit (Thermo Scientific Pierce ECL, USA). The blots were analyzed with a real-time chemiluminescence system (GE Healthcare, Sweden). β-actin was used as an internal loading control.

### Statistical analysis

GraphPad PrismTM 8.0 (GraphPad Software, USA) was used for statistical analysis. Values were reported as mean ± standard deviation (SD). Statistical differences of the behavioral results were evaluated by one-way analysis of variance (ANOVA) among the groups, while repeated measures ANOVA was used when analyzing the histological results. The post-hoc analysis was utilized by Tukey's multiple comparison test. A value of *p* < 0.05 was considered to be significant. Paired *t*-tests (two-tailed, α = 0.05) were used to compare the results of the contralateral and ipsilateral sonicated hemispheres.

## Results

### Assessment of BBB opening by FUS/MB

We first investigated the properties of MBs prepared in our laboratory. Figure 1C and 1D show representative images of a batch of freshly prepared MBs, which were polydispersed with a mean diameter of 1.5 μm and a concentration of 2.0×10^9^/mL. MBs with diameter less than 2.7 μm accounted for 90% in total. The signal intensity of the contrast-enhanced B-mode images of the brain rose to about 40 dB after MB administration (Figure 1E). The signal intensity gradually dropped to the half level about 7 min later and to the initial amplitude about 15 min later.

**Figure 2:**
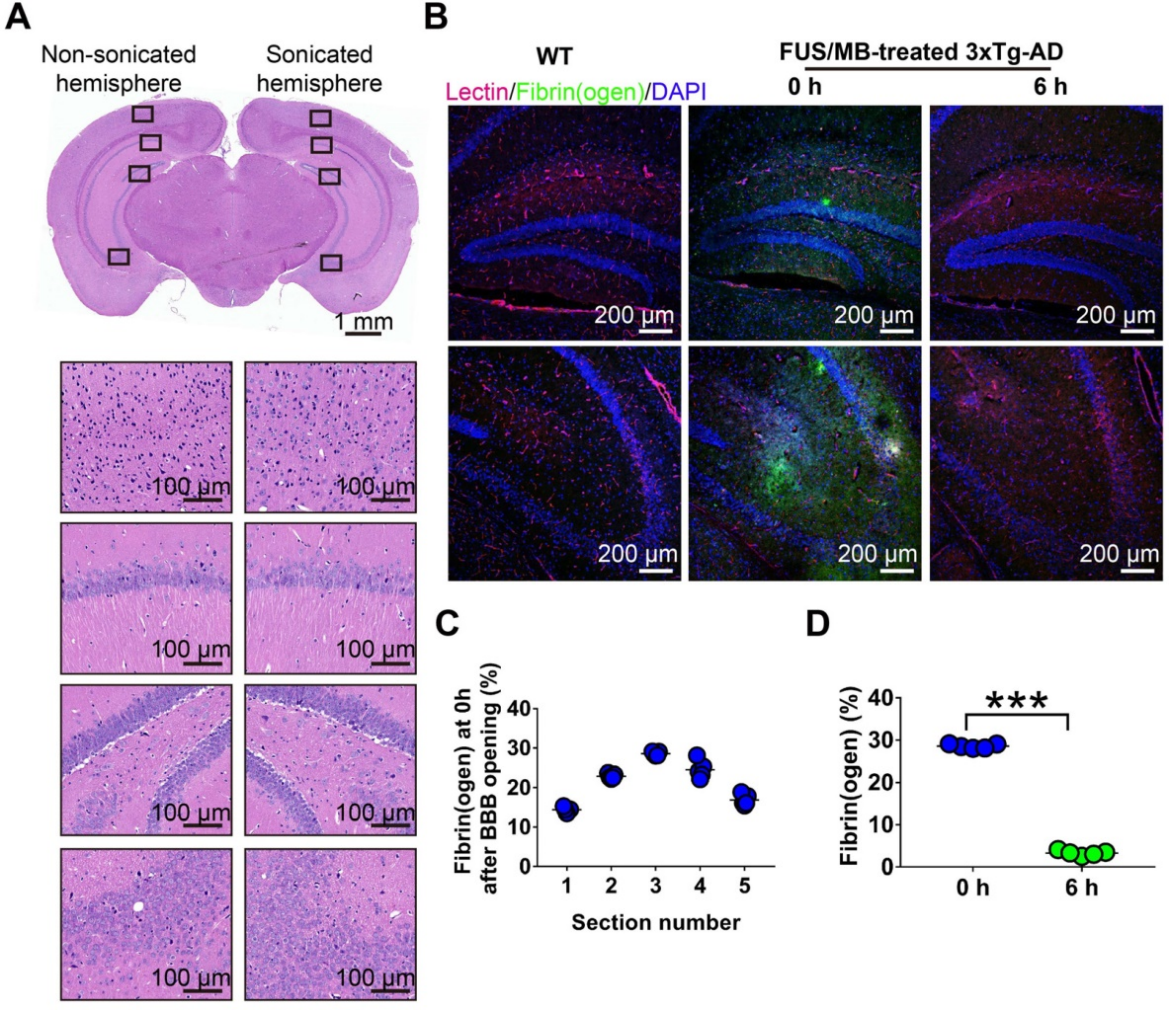
** Histological assessment of BBB opening induced by FUS/MB treatment in 3×Tg-AD mice.** (**A**) Representative microphotographs of a FUS/MB-treated brain stained with H&E. Upper left: the non-sonicated side. Upper right: the sonicated side. Lower left and right: images with high magnification in the black solid boxes. Upper scale bar: 1 mm, scale bars in the lower subfigures: 100 µm. There was neither hemorrhage nor erythrocyte extravasation nor vacuolations in the sonicated region. (**B**) Representative confocal fluorescence images of fibrin(ogen) leakage after BBB opening by FUS/MB. Images were acquired from the medial hippocampal sections in 3×Tg-AD mice immediately or at 6 h after BBB opening, as well as the WT mice. Scale bar: 200 µm. Red: lectin-positive microvessels. Green: extravascular fibrin(ogen). Blue: DAPI stained nuclei. (**C**) Quantification of extravascular fibrin(ogen) deposits in the hippocampus of 3×Tg-AD mice right after BBB opening. Five equally spaced sections (500 µm apart) were used in each animal (n = 5). The distribution of the immunopositive area across the coronal sections corresponded to the profile of the FUS pressure field laterally, with median sections showing the largest area. (**D**) Fibrin(ogen) positive area immediately and at 6 h after BBB opening by FUS/MB at the medial hippocampal section. At 6 h after the treatment, positive immunofluorescence signal drastically dropped. ***: *p* < 0.001.

To evaluate BBB opening by FUS/MB treatment, Evans blue dye (EB), which binds to albumin (molecular weight: 67 kDa) after entering the circulation, was injected intravenously with MBs. EB-tagged albumin extravasated from vasculature into the brain parenchyma only when the BBB was opened. With the parameters shown in Figure 1A, FUS/MB was able to successfully open the BBB with our MBs, as indicated by the blue coloration on the right sonicated hemisphere (Figure 1F). In contrast, no visible blue spots appeared in the unsonicated hemisphere contralaterally, as well as in the only FUS-treated hemisphere. This was also illustrated by fluorescence imaging of the same brain tissues (Figure 1G). Figure 2A depicts representative images of H&E stain of the FUS/MB treated brain, in which the right hemisphere was sonicated and the contralateral side was unsonicated. There was neither hemorrhage nor erythrocyte extravasation nor vacuolations in the sonicated region. As revealed by Nissl staining, no abnormalities in neuron integrity were discernible compared with the contralateral region (Figure S1A). Thus, these qualitative results indicated that no gross neuron or tissue damage occurred in the brain after FUS/MB treatment with our parameters. And the time course study showed BBB closure in 24 hours (Figure S1B-C).

In addition, we detected fibrinogen leakage right after the BBB was disrupted by FUS/MB (Figure 2B). The distribution of the immunopositive area across the coronal sections corresponded to the profile of the FUS pressure field laterally, with median sections showing the largest area (Figure 2C). However, at 6 h after the treatment, the positive immunofluorescence signal drastically dropped, indicating potential clearance of fibrinogen (Figure 2D).

### FUS/MB treatment improved learning and memory in 3×Tg-AD mice

#### Results of the Y-maze test

**Figure 3:**
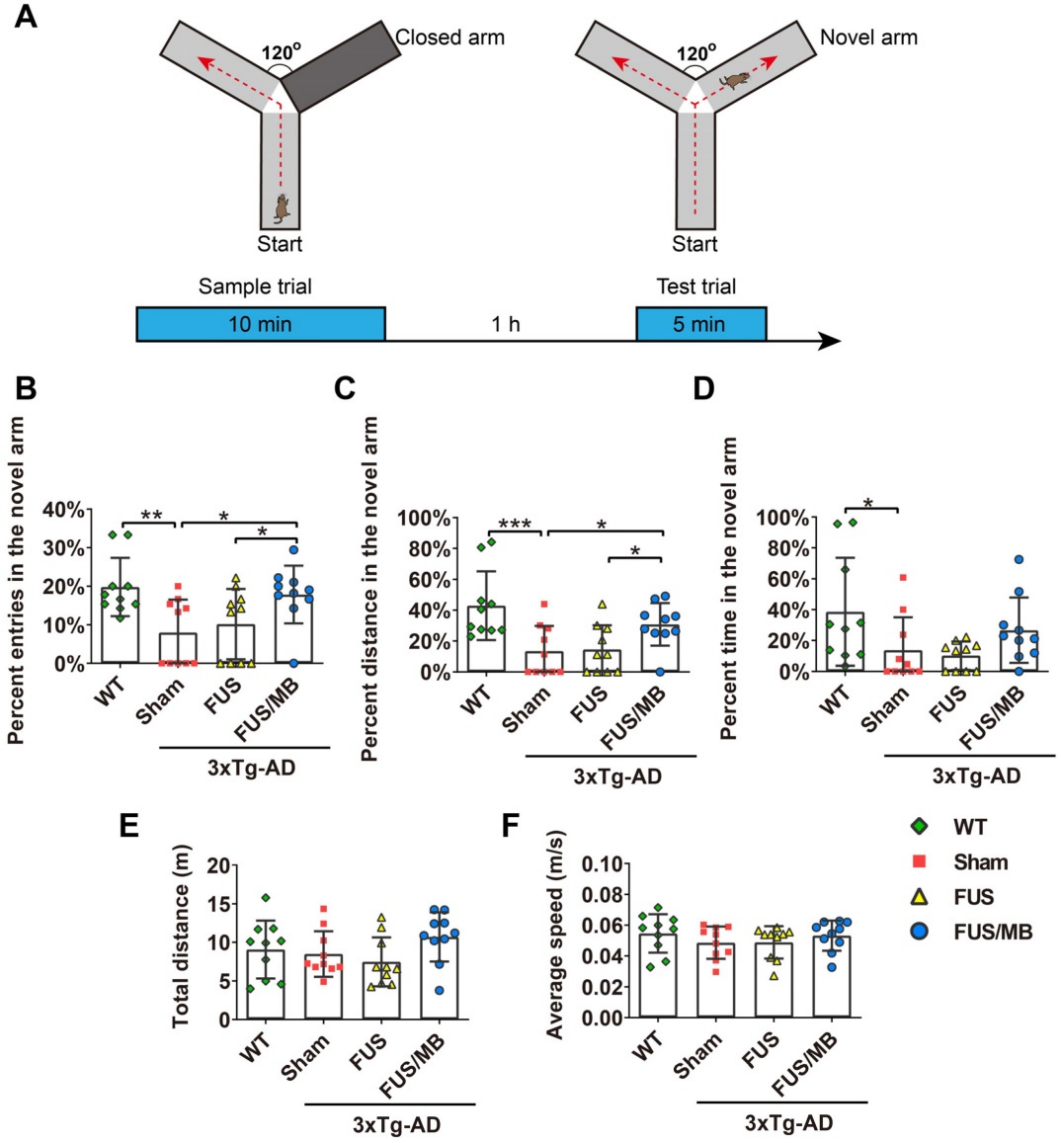
** The performance of the mice in the Y-maze test.** (**A**) Illustration of the Y-maze and the timeline of sample and test trial phases. (**B-D**) The percentage of entries, distance and time in the novel arm over all the arms in the WT, sham, FUS and FUS/MB group during the test trial phase. Following six weeks of FUS/MB treatment, the 3×Tg-AD mice showed significant increase in the percentage of entries into the novel arm (17.8% ± 7.4%) by 2.2-fold compared with the sham group and 1.7-fold (8.0% ± 8.0%, *p* < 0.05) compared with the FUS group (10.2% ± 9.1%, *p* < 0.05). The average percentage of the distance traveled in the novel arm of the FUS/MB group was 30.8% ± 13.8%, which was 2.3 times and 2.1 times that of the sham group (13.4% ± 16.3%, *p* < 0.05) and FUS group (14.5% ± 15.6%, *p* < 0.05), respectively. Albeit with no significance, the FUS/MB group showed on average, prolonged time in the novel arm (26.6% ± 21.2%), compared with the sham-treated (13.7% ± 21.2%, *p* =0.227) and FUS-treated (10.2% ± 9.1%, *p* = 0.126) 3×Tg-AD mice. (**E-F**) Total distance and the average speed of the animals traveled in all the arms. Statistical analysis revealed no significant differences in the total distance and average speed between groups, indicating no motor impairments in the 3×Tg-AD mice. n = 10 for each group. Data were presented as mean ± SD. *: *p* < 0.05, **: *p*<0.01, ***: *p*< 0.001.

Y-maze was used to assess short-term working memory of the animals in the present study (Figure 3). During the test trial, the 3×Tg-AD mice in the sham group showed significantly fewer entries into the novel arm (8.0% ± 8.0%), entering only half as many times as the WT mice (19.8% ± 7.5%, *p* < 0.01, Figure 3B). The percentage of the distance they travelled in the novel arm showed similar results, with the sham group traveling 13.4% ± 16.3% and the WT group 42.9% ± 22.2% (*p* < 0.01, Figure 3C). The 3×Tg-AD mice spent an average of 13.8% ± 21.2% of the total time exploring the novel arm, compared with 38.6% ± 34.9% spent by the WT mice (*p* < 0.05, Figure 3D). The data were indicative of impaired spatial working memory of the 3×Tg-AD mice compared with the WT mice. Notably, following FUS/MB treatment, the 3×Tg-AD mice showed significant increase in the percentage of entries into the novel arm (17.8% ± 7.4%) by 2.2-fold compared with the sham group (8.0% ± 8.0%, *p* < 0.05) and 1.7-fold compared with the FUS group (10.2% ± 9.1%, *p* < 0.05). The average percentage of the distance traveled in the novel arm by the FUS/MB group was 30.8% ± 13.8%, which was 2.3 times and 2.1 times that of the sham group (13.4% ± 16.3%, *p* < 0.05) and FUS group (14.5% ± 15.6%, *p* < 0.05), respectively. Albeit with no significance, the FUS/MB group showed on average, prolonged time in the novel arm (26.6% ± 21.2%), compared with the sham-treated (13.7% ± 21.2%, *p* = 0.227) and FUS-treated (10.2% ± 9.1%, *p* = 0.126) 3×Tg-AD mice. The 3×Tg-AD mice in the FUS group did not show significant differences from the sham group. Statistical analysis revealed no significant differences in the total distance and average speed between groups, indicating no motor impairments in the 3×Tg-AD mice (Figure 3E and 3F).

#### Results of the Morris water maze test

**Figure 4:**
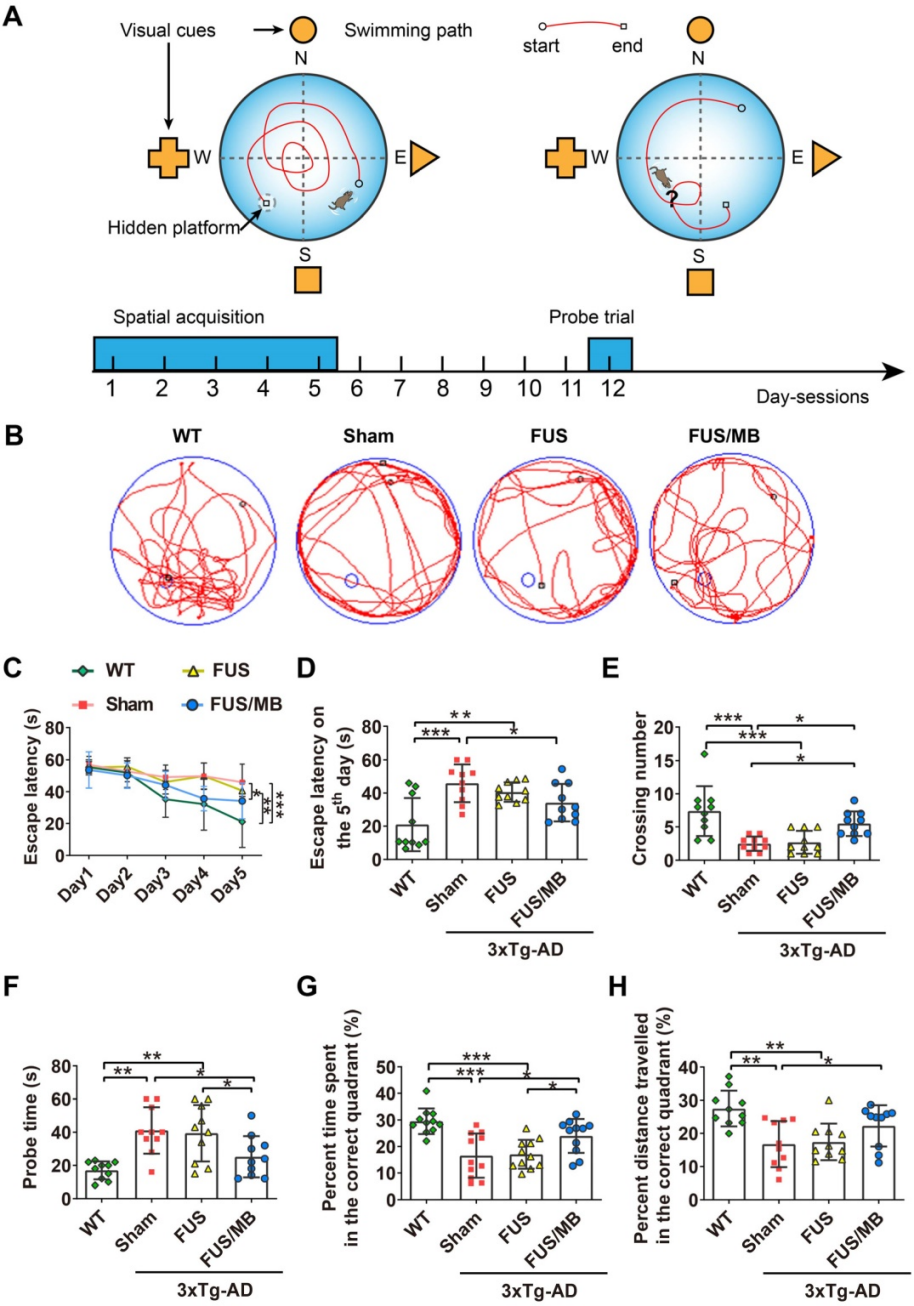
** The performance of the mice in the Morris water maze test.** (**A**) Illustration of the Morris water maze and the timeline of spatial acquisition and probe trial sessions. (**B**) Representative swimming paths of the 3×Tg-AD mice receiving sham, FUS or FUS/MB treatment and non-transgenic littermates (WT) during the probe trial. (**C-D**) The escape latencies in the four groups over five consecutive training days (C) and on the 5th day of the spatial acquisition session (D). Comparing with the WT mice, sham-treated or FUS-treated 3×Tg-AD mice showed learning impairment. The escape latency for FUS/MB-treated 3×Tg-AD mice was 34.2 ± 11.2 s, while 45.9 ± 11.4 s for the sham group (*p* < 0.05) and 40.6 ± 5.8 s for the FUS group (*p* = 0.112). (**E-F**) The average crossing number over the platform-site and the latency of the first target-site crossover (probe time) during the probe trial. The average crossing number of the FUS/MB group was an average of 5.5 ± 1.8, 2.7-fold that of the sham group (*p* < 0.05) and 2.0-fold that of the FUS group (2.7 ± 1.7, *p* < 0.05). Regarding the probe time, the WT group spent 17.0 ± 5.4 s to first reach the platform-site, while the sham group took 41.1 ± 14.0 s (*p* < 0.001) and the FUS group took 39.4 ± 17.0 s (*p* < 0.01). In contrast, the FUS/MB group (25.2 ± 12.4 s) took 38% and 36% less time compared with the sham group (*p* < 0.05) and the FUS group (*p* < 0.05). (**G-H**) The percentage of time spent and distance traveled in the target quadrant during the probe trial. The percent time and distance in the correct quadrant showed considerable increase in the FUS/MB group, by 45% (24.0% ± 6.4% vs. 16.6% ± 8.3%, *p* < 0.05) and 33% (22.3% ± 2.0% vs. 16.8% ± 2.2%, *p* < 0.05), respectively, compared with the sham group. n = 10 for each group. Data were presented as mean ±SD. *: *p* < 0.05, **: *p* < 0.01, ***: *p* < 0.001.

Our study assessed spatial learning and long-term working memory was assessed using the Morris water maze test. During the spatial acquisition period, the escape latency, which was the time the mouse took to find the hidden platform, was measured (Figure 4C and 4D). For WT mice, the escape latency decreased over five consecutive training days, with the value on the fifth day (21.0 ± 16.0 s) only 38% of the value on the initial day (55.3 ± 3.6 s). Comparatively, for the sham group on the fifth day, it still took 82% of the initial latency on the first day (56.9 ± 5.3 s) to find the hidden platform (45.9 ± 11.4 s), which was 2.2-fold of the time of the WT group (*p* < 0.001), indicating learning impairment in the sham-treated 3×Tg-AD mice. In the FUS only group, the escape latency showed a similar trend to that of the sham group, and was 1.9-fold that of the WT group (*p* < 0.01). Evidently, the escape latency of the 3×Tg-AD mice treated with FUS/MB on the last training day decreased to 64% of the escape latency on the first day, with a value of 34.2 ± 11.2. The escape latency was 45.9 ± 11.4 s for the sham group (*p* < 0.05) and 40.6 ± 5.8 s for the FUS group (*p* = 0.112).

The probe trial was performed to assess the long-term memory of the animals six days after the training session. The swimming path of WT mice showed a pattern of target scanning, which focused on regions surrounding the platform (Figure 4B). However, the trajectory pattern of the sham and FUS groups were characterized by thigmotaxis and random search. The FUS/MB-treated mice started to swim inwards and then made concentric paths across the platform site, indicating that the mice had memorized the distance from the walls to the platform. There was no significant difference in all the quantitative indices between the FUS group and sham group. The average crossing number over the platform-site of the sham group was 2.5 ± 1.1, only one third that of the non-transgenic littermates (7.4 ± 3.7, *p* < 0.001), while that of the FUS/MB group was an average of 5.5 ± 1.8, 2.7-fold that of the sham group (*p* < 0.05) and 2.0-fold that of the FUS group (2.7 ± 1.7, *p* < 0.05) as shown in Figure 4E. Regarding the probe time (Figure 4F), the WT group spent 17.0 ± 5.4 s to first reach the platform-site, while the sham group took 41.1 ± 14. s (*p* < 0.001) and the FUS group took 39.4 ± 17.0 s (*p* < 0.01). In contrast, the FUS/MB group (25.2 ± 12.4 s) took 38% and 36% less time compared with the sham group (*p* < 0.05) and the FUS group (*p* < 0.05). Likewise, the percentage of the time spent and the distance travelled in the correct quadrant showed considerable increase in the FUS/MB group, by 45% (24.0% ± 6.4% vs. 16.6% ± 8.3%, *p* < 0.05) and 33% (22.3% ± 2.0% vs. 16.8% ± 2.2%, *p* < 0.05), respectively, compared with the sham group (Figure 4G and 4H).

#### Results of the step-down passive avoidance test

**Figure 5:**
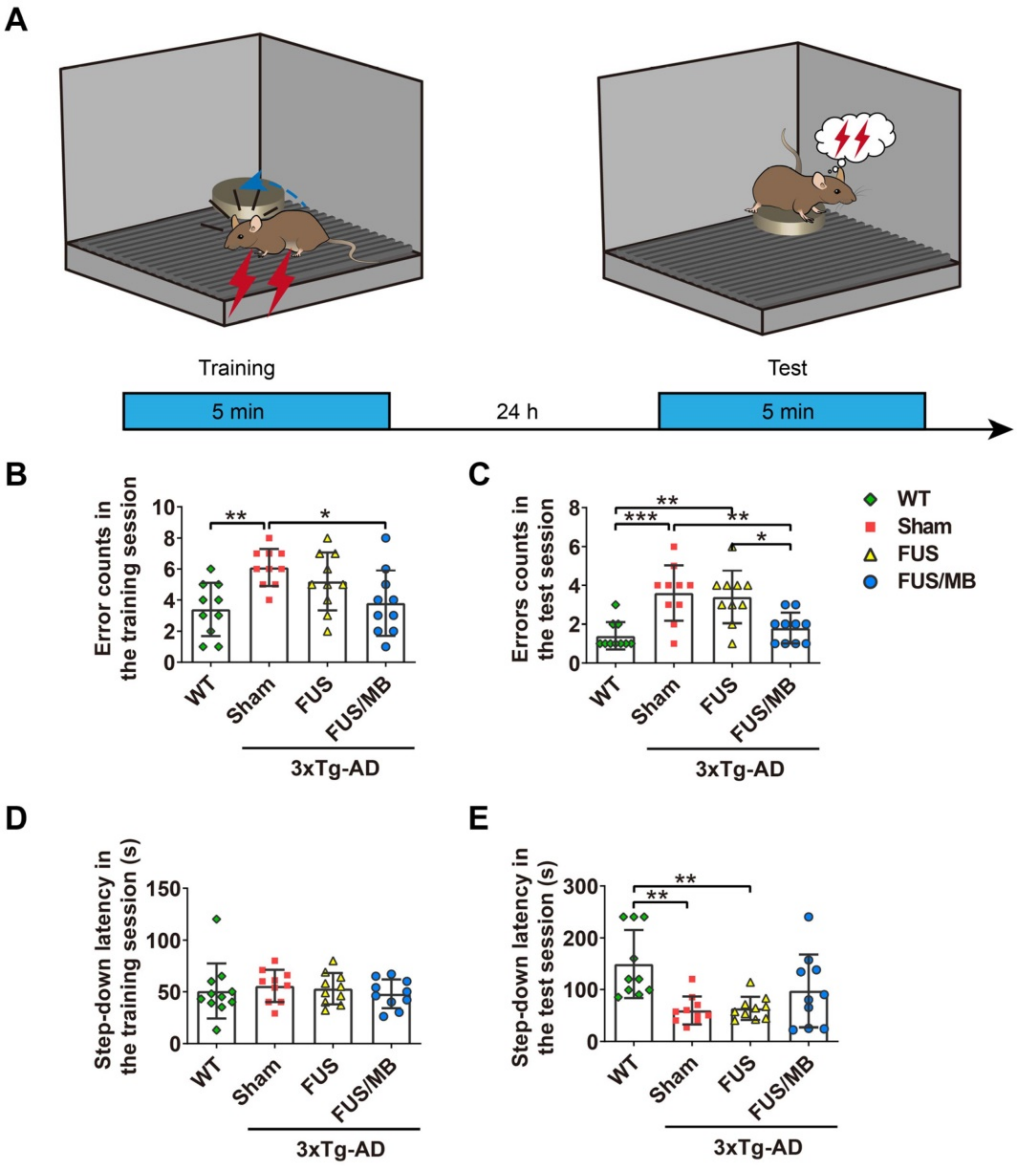
** The performance of the mice in the step-down passive avoidance test.** (**A**) Illustration of the step-down passive avoidance test and the timeline of the training and test session. (**B-C**) The number of the times of the mice stepped down from the platform onto the grid (error counts) in the WT, sham, FUS and FUS/MB groups during the training (B) and the test session (C). In the test phase, the error counts of the FUS/MB group (1.8 ± 0.8) showed evident improvement, with significantly fewer errors than those of the sham group (3.6 ± 1.4, *p* < 0.01) and the FUS group (3.4 ± 1.3, *p* < 0.05). (**D-E**) The step-down latency, the time it took the mouse to step down from the platform onto the grid, of the animals in the four groups during the training (D) and the test session (E). In the training session, there were no significant differences in the latencies between groups. In the test session, the WT mice showed an evidently prolonged latency (149.3 ± 66.0 s) while the sham-treated group (59.5 ± 27.0 s) and FUS-treated 3×Tg-AD mice (63.5 ± 22.5 s) did not. Albeit without significance, the step-down latency of the FUS/MB group showed a 64% increase in comparison to the sham group (97.5 ± 70.4 s vs. 59.5 ± 27.0 s, *p* = 0.106) during the test phase, while the FUS group did not show this increase. n =10 for each group. Data were presented as mean ± SD. *: *p* < 0.05, **: *p* < 0.01, ***: *p* < 0.001.

The step-down latency and error counts were used as measurements of memory retention in this study (Figure 5B-5E). Compared with the WT mice, the sham-treated 3×Tg-AD mice exhibited a poor performance, represented by more errors in both the training (6.1 ± 1.2 vs. 3.4 ± 1.7, *p* < 0.01, Figure 5B) and test phases (3.6 ± 1.4 vs. 1.4 ± 0.7, *p* < 0.001, Figure 5C). In the training phase, there were no significant differences in the latencies between groups, within a range from 48 to 53 s (Figure 5D). In the test phase, the error counts of the FUS/MB group (1.8 ± 0.8) held a trend of improvement, with significantly fewer errors than those of the sham group (3.6 ± 1.4, *p* < 0.01) and the FUS group (3.4 ± 1.3, *p* < 0.05, Figure 5C). The WT mice showed an evidently prolonged latency (149.3 ± 66.0 s) while the sham-treated group (59.5 ± 27.0 s) and FUS-treated 3×Tg-AD mice (63.5 ± 22.5 s, Figure 5E) did not. Albeit without significance, the step-down latency of the FUS/MB group showed a 64% increase in comparison to the sham group (97.5 ± 70.4 s vs. 59.5 ± 27.0 s, *p* = 0.106) during the test phase, while the FUS group did not show this increase.

Together, the results of the three independent memory-related behavioral tests provided evidence that the repeatedly FUS/MB-treated 3×Tg-AD mice had significantly improved performance in both short-term and long-term memory, as well as cognition, suggesting that FUS/MB treatment effectively rescued the spatial memory deficits in eight-month-old 3×Tg-AD mice. The FUS group that received the same sonication procedure, but without MBs, showed poor behavior, similar to the sham group.

### Results of histological analysis

Mice in each group were sacrificed three days later after the completion of all the behavioral tests for histological analysis. After cardiac perfusion, their brains were excised and processed for immunostaining.

#### FUS/MB treatment ameliorated Aβ load in 3×Tg-AD mice

**Figure 6:**
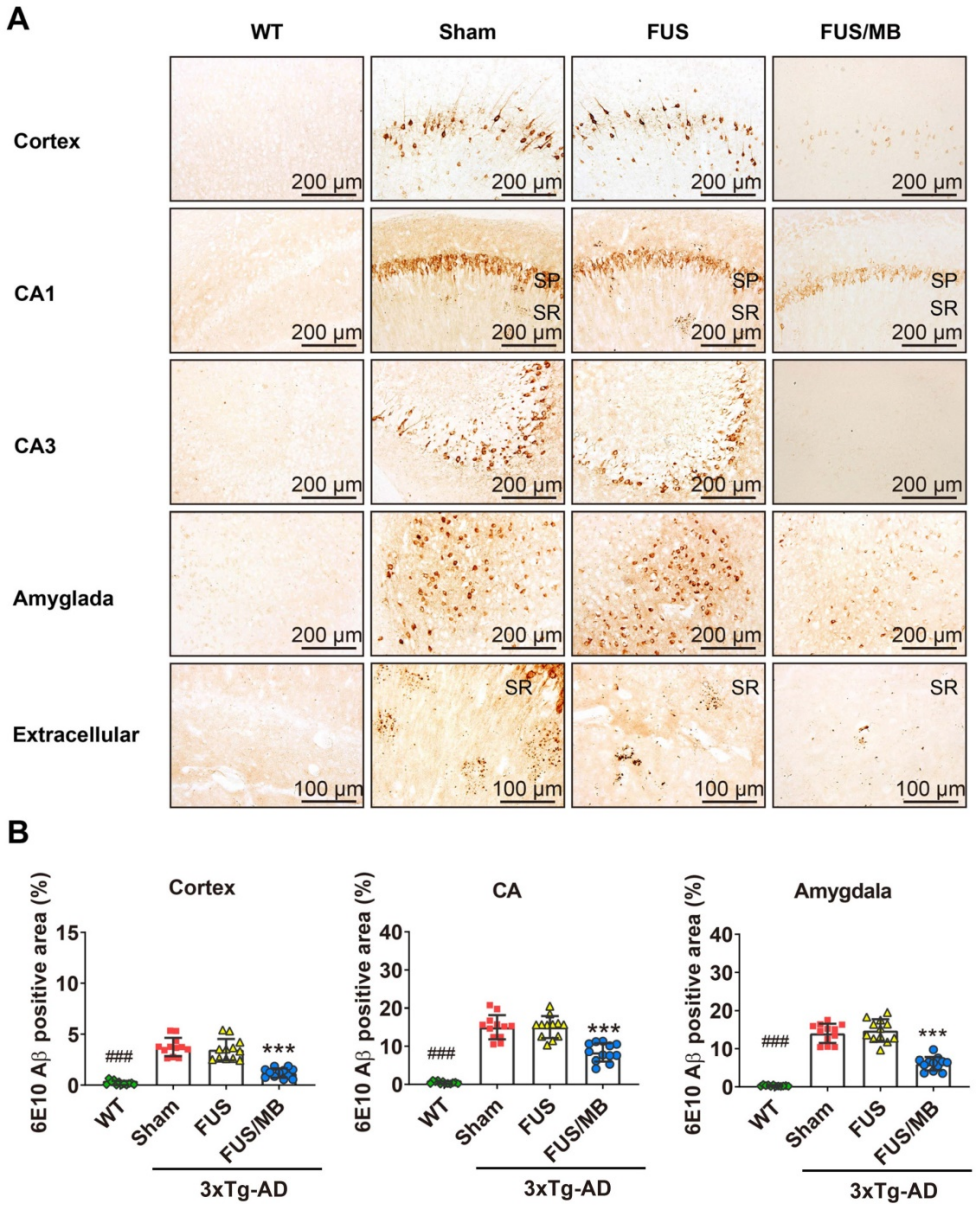
** Aβ pathology in the cortex, hippocampus, and amygdala of the brain sections from 3×Tg-AD mice in the sham, FUS and FUB group, as well as the WT mice. Coronal sections were stained with anti-Aβ antibody 6E10.** (**A**) Representative immunohistochemical images of the sonicated hemisphere in the FUS/MB group and ipsilateral sides in other three groups. Intraneuronal Aβ immunoreactivity became prominent in the cortex, CA1 and CA3 pyramidal cells (SP) of the hippocampal formation, and the amygdala in sham-treated and FUS-treated 3×Tg-AD mice. Abundant extracellular diffuse Aβ protofibrillar deposits were observed predominantly in the stratum radiatum (SR) of the hippocampus. Reduced Aβ pathology in the cortex, CA1 and CA3 subfield, as well as in the amygdala, could be observed in the FUS/MB-treated 3×Tg-AD mice compared with the sham and FUS-treated group. (**B**) Quantitative analysis of 6E10-positive areas in the cortex, CA region and amygdala of the ipsilateral hemispheres in the four groups. The 6E10-positive areas in the cortex, CA and amygdala of the sham-treated 3×Tg-AD mice were 3.7% ± 0.9%, 15.0% ± 3.2%, and 14.1% ± 2.5%, which were reduced to 1.2% ± 0.5%, 8.4% ± 2.4%, and 6.1% ± 1.8% after FUS/MB treatment, representing reductions of 67%, 44%, and 57%, respectively. There was no difference between the sham and FUS groups. ***: *p* < 0.001, vs. WT or sham or FUS. ###: *p* < 0.001, vs. sham or FUS or FUS/MB.

In the present study, plaque load within the hippocampus and cortex were examined with anti-Aβ 6E10, which recognizes amino acid residue 1-16 of Aβ 35. Intraneuronal Aβ immunoreactivity became prominent in the cortex, as well as CA1 and CA3 pyramidal cells of the hippocampal formation and amygdala in the sham and FUS groups (Figure 6A). Immunoreactive staining was pronounced within the soma of many neurons. Abundant shrunken neurons were distributed in the CA1 and CA3 subfield, and the amygdala. Although without obvious mature amyloid plaques, abundant extracellular diffuse Aβ deposits were observed predominantly in the stratum radiatum (SR) of the hippocampus (Figure 6A). These pathological results indicated that the 3×Tg-AD mice we used in the present study developed both intraneuronal and extracellular Aβ deposits.

For the FUS/MB-treated 3×Tg-AD mice, reduced Aβ pathology in the cortex, CA1 and CA3 subfield, as well as in the amygdala, could be observed compared with the sham and FUS groups. Moreover, the extracellular Aβ deposits in the SR layer of the FUS/MB-treated mice appeared markedly fewer than the sham-treated and FUS-treated mice. Further quantitative analysis showed that the 6E10 positive areas in the cortex, CA, and amygdala of the sham-treated 3×Tg-AD mice were 3.7% ± 0.9%, 15.0% ± 3.2%, and 14.1% ± 2.5%, which were reduced to 1.2% ± 0.5%, 8.4% ± 2.4%, and 6.1% ± 1.8% after FUS/MB treatment, representing reductions of 67%, 44%, and 57%, respectively (*p* < 0.001, Figure 6B). There was no difference between the sham and FUS groups. Comparisons of Aβ pathology in the contralateral sides of hemispheres between the FUS/MB and sham group showed no significant difference emerging from the unilateral FUS/MB treatments (Figure S5).

The examination of Aβ pathology was also conducted on the contralaterally non-sonicated and sonicated hemispheres from the same brain section (Figure S2). Scanning of the whole brain sections was performed. Distinction of 6E10 immunoreactivity between the non-sonicated and sonicated hemispheres was observed in the cortex, CA region, and amygdala, as shown in Figure S2A. The 6E10 positive areas in the cortex, CA, and amygdala of the sonicated side from medial brain sections reduced at the order of 72%, 43%, and 58% compared to the contralaterally non-sonicated side (*p* < 0.001, paired *t*-test, Figure S2B).

These observations suggested that FUS/MB effectively ameliorated the Aβ deposits in 3×Tg-AD mice. In particular, unilateral FUS/MB treatment on the right hemisphere reduced Aβ load compared with the contralateral side.

#### FUS/MB treatment reduced tau phosphorylation in 3×Tg-AD mice

**Figure 7:**
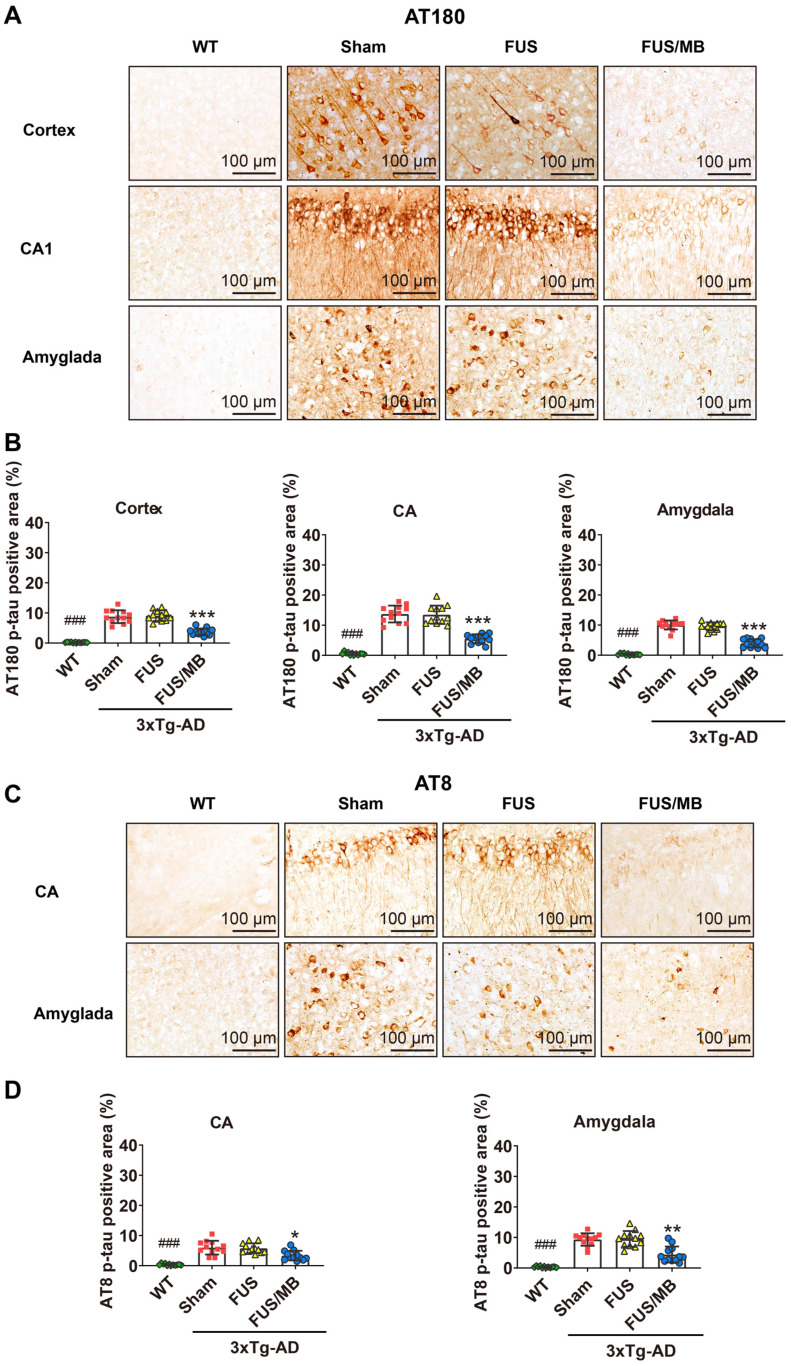
** Phosphorylated tau stained with AT180 or AT8 in the brain sections from 3×Tg-AD mice in the sham, FUS and FUS/MB group, as well as the WT mice.** (**A**) Representative immunohistochemical images against AT180 of the sonicated hemisphere in the FUS/MB group and ipsilateral sides in other three groups. AT180 p-tau pathology was evident in the cortex, hippocampus and amygdala of the sham-treated and FUS-treated 3×Tg-AD mice. Notably, the FUS/MB group showed reductions in the AT180-immunoreactive signals. Scale bar: 100 µm. (**B**) Quantitative analysis of AT180-positive areas in the cortex, CA region and amygdala of the ipsilateral hemispheres in the four groups. The AT180-positive areas of the sham group in the cortex, CA region and amygdala were 8.7% ± 2.1%, 13.7% ± 2.8%, and 10.1% ± 1.5%, which were reduced to 3.7% ± 1.1%, 5.5% ± 1.5%, and 4.0% ± 1.4% after FUS/MB treatment, representing reductions of 57%, 60%, and 60%, respectively. There was no difference between the sham and FUS groups. (**C**) Representative immunohistochemical images against AT8 of the sonicated hemisphere in the FUS/MB group and ipsilateral sides in other three groups. AT8 p-tau pathology was evident in the hippocampus and amygdala of the sham-treated and FUS-treated 3×Tg-AD mice. FUS/MB treatment also induced amelioration of the AT8-posoitive signals. Scale bar: 100 µm. (**D**) Quantitative analysis of AT8-positive areas in the CA region and amygdala of the ipsilateral hemispheres in the four groups. AT8-immunoreactive areas were decreased in CA and amygdala of the 3×Tg-AD mice after FUS/MB treatments by orders of 45% and 53% compared with the sham group. There was no difference between the sham and FUS groups. Three brain sections per animal were used. *, **, ***: *p* < 0.05, <0.01, < 0.001, vs. WT or sham or FUS. ###: *p* < 0.001, vs. sham or FUS or FUS/MB.

Tau phosphorylation was assessed with anti-tau AT180 and anti-tau AT8. AT180 detects tau phosphorylation at the Ser235 and Thr231 sites, an early event in the assembly of tau into filaments 36. AT8 marks tau phosphorylation at both Ser202 and Thr205, and is an antibody widely used to stage the progression of tau pathology in relation to neurofibrillary tangle formation 36, 37. The phosphorylated tau stained with AT180 (Figure 7A) and AT8 (Figure 7C) was detectable in both the soma and axon of the pyramidal cells in the CA1 subfield of the hippocampus in the sham and FUS groups. The staining of axonal projections extended into the SR layer of the hippocampus. AT180- and AT8-positive neurons were also found in the amygdala. Moreover, the AT180-positive area in the CA region was 2.3-fold that of the AT8-positive area for the sham-treated 3×Tg-AD mice, as well as the FUS-treated mice (Figure 7B and 7D). Additionally, in the cortex, we found AT180-positive neurons with axonal p-tau (Figure 9A and 10A), which were not found for AT8. These results confirmed that the 3×Tg-AD mice we used in the present study developed tau pathology concomitant with Aβ load.

For the FUS/MB-treated 3×Tg-AD mice, mitigating AT180 p-tau signal was observed in the cortex, hippocampus and amygdala (Figure 7A). The AT180-positive areas of the sham group in the cortex, CA region and amygdala were 8.7% ± 2.1%, 13.7% ± 2.8%, and 10.1% ± 1.5%, which were reduced to 3.7% ± 1.1%, 5.5% ± 1.5%, and 4.0% ± 1.4% after FUS/MB treatment, representing reductions of 57%, 60%, and 60%, respectively (*p* < 0.001, Figure 7B). Similarly, the AT8-immunoreactive areas were decreased in the CA and amygdala of the 3×Tg-AD mice after FUS/MB treatments by orders of 45% and 53% when compared with the sham group (Figure 7C-D). There was no difference between the sham and FUS groups.

The examination of p-tau pathology on the contralaterally non-sonicated and sonicated hemispheres from the same section showed that FUS/MB treatment resulted in amelioration of tau phosphorylation (Figure S3-S4). For the AT180-positive areas, reductions in the cortex, CA, and amygdala of the sonicated side were reduced by orders of 57%, 60%, and 61% compared with the contralaterally non-sonicated side (*p* < 0.001, paired *t*-test, Figure S3B). For the AT8-positive areas, unilateral FUS/MB resulted in reductions of 44% and 53% in the CA and amygdala of the sonicated hemisphere compared with the contralateral side (Figure S4).

These results implied that the FUS/MB treatment induced a positive effect in ameliorating tau phosphorylation in eight-month-old 3×Tg-AD mice. Comparing to the contralaterally non-sonicated hemisphere, remarkable attenuation of tau phosphorylation pathology was noted in the sonicated side induced by unilateral FUS/MB treatment.

#### FUS/MB treatment improved hippocampal neuron axonal health in 3×Tg-AD mice

**Figure 8:**
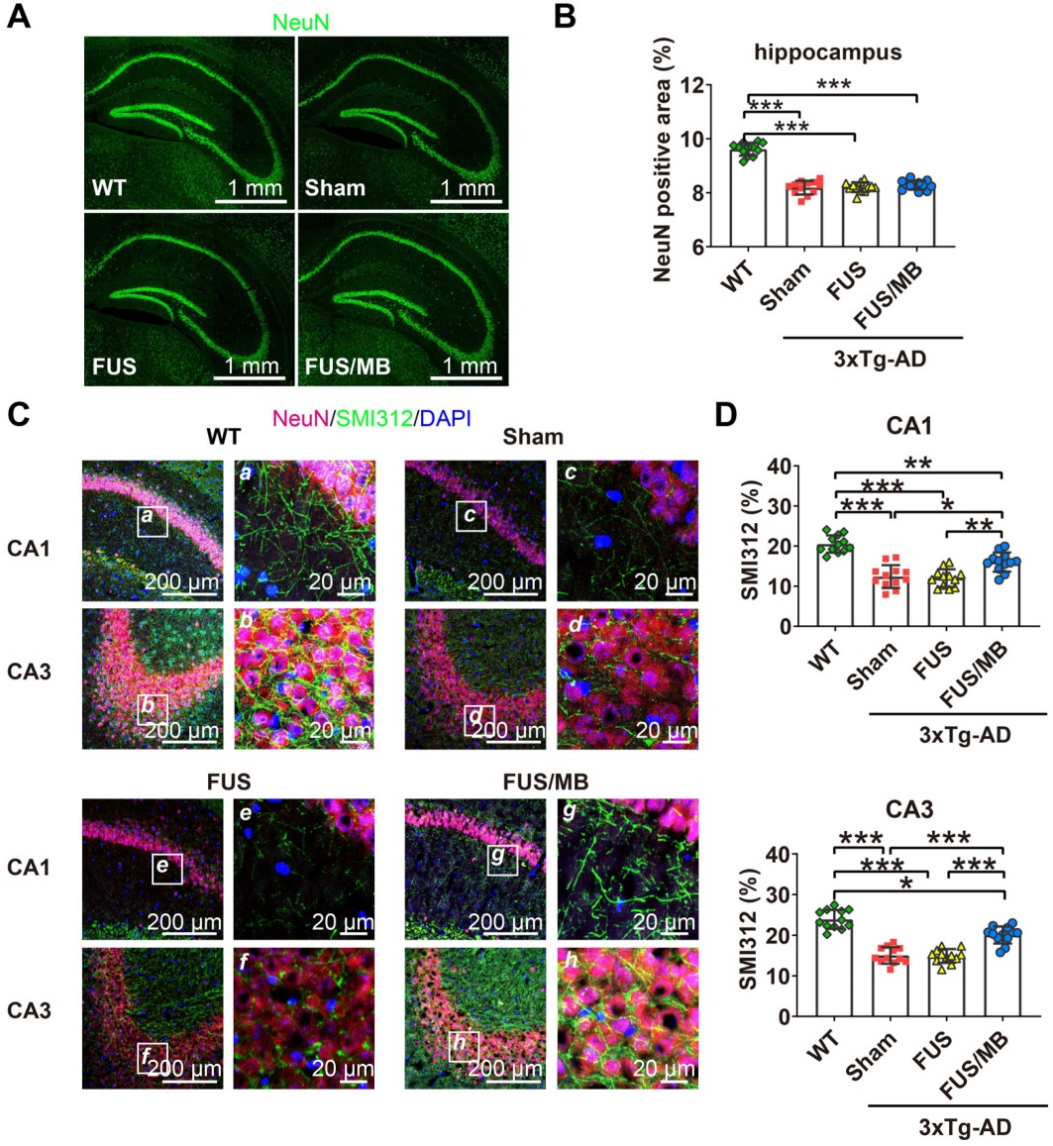
** Neuronal health in the hippocampus of coronal brain sections from the WT, sham, FUS and FUS/MB group. Anti-NeuN and anti-SMI312 antibodies were used.** (**A**) Representative stitched confocal fluorescence images of NeuN-positive neurons in the hippocampus of the sonicated hemisphere in the FUS/MB group and ipsilateral sides in other three groups. NeuN: green. Scale bar: 1 mm. (**B**) NeuN positive areas in the hippocampus of the ipsilateral sides in the four groups. Quantitative analysis showed reduced NeuN-positive areas in the sham-treated and FUS-treated 3×Tg-AD mice compared with the WT mice (*p* < 0.001). No significant differences emerged from the FUS/MB treatment when comparing with the sham and FUS group. (**C**) Axonal neurofilament in the hippocampus of coronal brain sections from the sonicated hemisphere in the FUS/MB group and ipsilateral sides in other three groups. Brain sections were counterstained with the anti-NeuN (red) and anti-SMI312 (green) antibodies. Representative confocal fluorescence images with low magnification in CA1 and CA3 subregions of the four groups were showed in the left columns; Scale bar: 200 µm. Right columns (a-h): Enlarged images from the white boxes in the left images; Scale bar: 20 µm. (**D**) Quantitative analysis of SMI312-positive areas in the four groups. Axonal neurofilament degeneration of the 3×Tg-AD mice in the sham and FUS group was indicated by the poor SMI312-positive signal in the CA1 and CA3 subregions, compared with the WT group (*p* < 0.001). Significant improvement was detected in the hippocampus of the FUS/MB group, enhancing by orders of 29% (*p* < 0.05) for the SMI312-positive area in CA1 and 33% (*p* < 0.001) in CA3 subregion compared with the sham group. Three brain sections per animal were used. *: *p* < 0.05; **: *p* < 0.01; ***: *p* < 0.001, with n = 4 per group.

Upon observation of the reduction in Aβ and phosphorylated tau pathology from the sonicated hippocampi in the FUS/MB group, we sought to examine the neuronal health through neuronal and axonal neurofilament staining (Figure 8A). Compared with the WT group (9.6% ± 0.2%), 3×Tg-AD mice in the sham and FUS groups showed neuronal compromise, with NeuN-positive areas of 8.2% ± 0.3% (*p* < 0.001) and 8.2% ± 0.2% (*p* < 0.001) in the hippocampus respectively (Figure 8B). In the FUS/MB group, no significant differences in the NeuN-positive area emerged from the treatment.

In addition, axonal health was immunolabeled using an antibody against phosphorylated neurofilament proteins (SMI312), which are the major components of the neuronal cytoskeleton and provide axonal support. Confocal microscopy analysis indicated axonal neurofilament degeneration by the poor SMI312-positive signal in the hippocampus, for instance, in the CA1 and CA3 sections of the 3×Tg-AD mice in the sham and FUS groups compared with the WT group (Figure 8C). Conspicuously, significant improvement was detected in the hippocampus of the FUS/MB group, enhancing by orders of 29% (*p* < 0.05) and 33% (*p* < 0.001) for the SMI312-positive area in CA1 and CA3 compared with the sham group (Figure 8D).

#### FUS/MB treatment activated Aβ internalization by microglia in 3×Tg-AD mice

**Figure 9:**
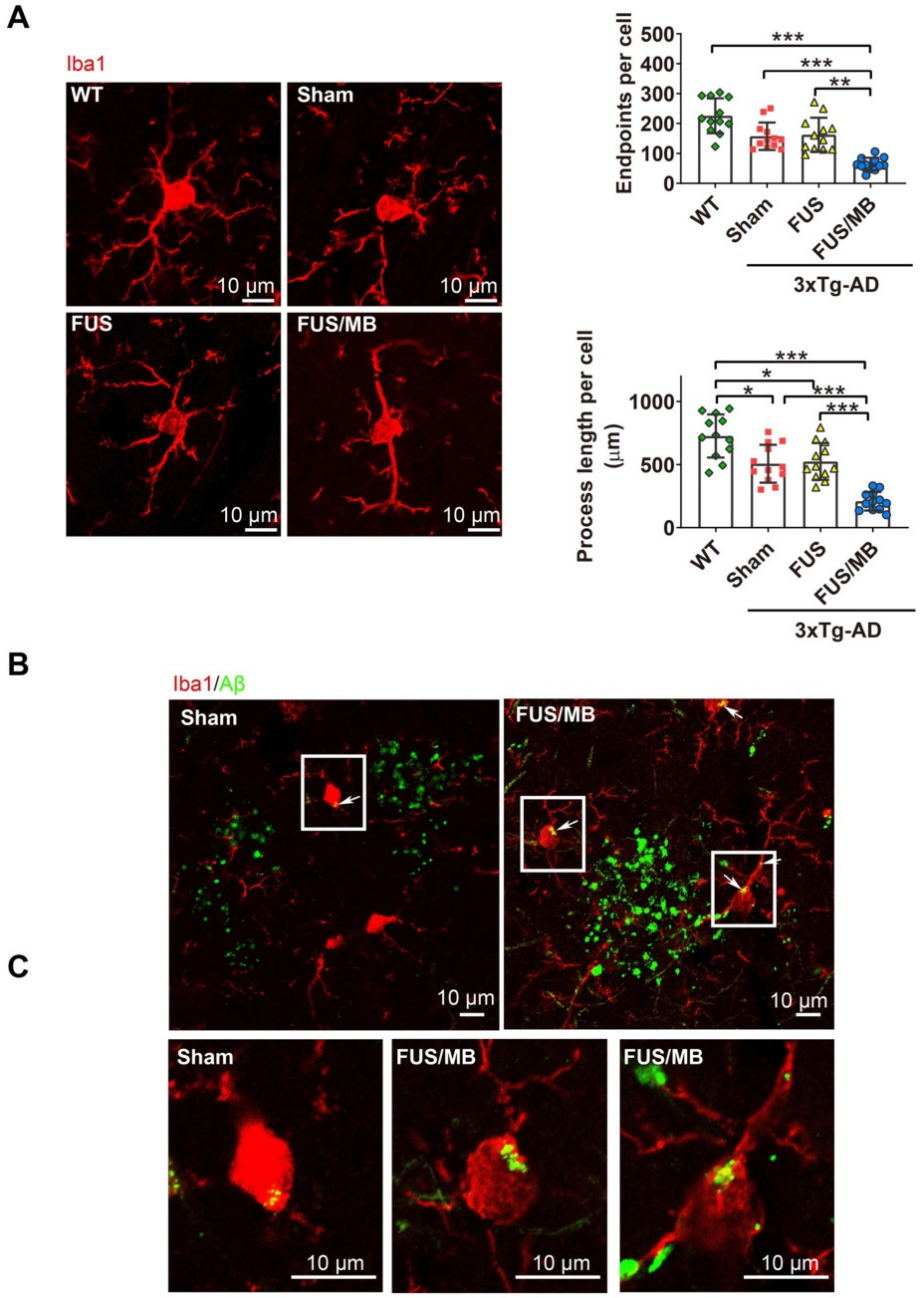
** Confocal microscopic analysis of microglial activation and phagocytosis of Aβ deposits induced by FUS/MB treatment in the 3×Tg-AD mice.** (**A**) Representative confocal fluorescence images of microglial cells in ipsilateral hippocampi of the 3×Tg-AD mice receiving sham, FUS and FUS/MB treatment, as well as the WT mice. The brain sections were immunostained using the microglial cytoplasmic marker Iba1 (Red). Scale bar: 10 µm. Resting microglial cells in the hippocampus of the WT mice displayed a healthy morphology with highly ramified processes while microglia in the sham-treated and FUS-treated 3×Tg-AD mice showed slightly fewer branched processes, indicating inherently affected by the pathology. Notably, profound morphological alteration of microglia with reduced branches could be observed in the hippocampus of FUS/MB-treated 3×Tg-AD mice where extracellular Aβ deposits existed. The quantification revealed that microglia in the FUS/MB group were more activated as reflected by reductions of 59% in the endpoints (*p* < 0.001) and 58% in the process length (*p* < 0.001) compared with the sham group. (**B**) Phagocytosis of Aβ deposits (white arrows) by microglia in the sham ipsilateral side and sonicated side in the FUS/MB group. Few microglia engulfing Aβ were observed in the sham group. However, following the application of FUS/MB, internalization of Aβ deposits by microglia were extensive surrounding the depositions. (**C**) Images of microglia with high magnification in the white boxes. Scale bar: 10 µm. *: *p* < 0.05; **: *p* < 0.01; ***: *p* < 0.001, with n = 4 per group.

We next sought to assess whether the microglia, as the brain-residing immune cells, were activiated by the FUS/MB-induced BBB opening. Brain sections were immunostained using the microglial cytoplasmic marker Iba1 (Figure 9A). Then skeleton analysis of microglia in the four groups was performed, in which microglial process endpoints and process length were normalized per cell (Figure 9B). Resting microglial cells in the hippocampus of the WT mice displayed a healthy morphology with highly ramified processes while microglia in the sham-treated and FUS-treated 3×Tg-AD mice showed slightly fewer branched processes, indicating inherently affected by the pathology (Figure 9A). Notably, profound morphological alteration of microglia with reduced branches could be observed in the SR layer of the hippocampus of FUS/MB-treated 3×Tg-AD mice where extracellular Aβ deposits existed. The quantification revealed that microglia in the FUS/MB group were more activated as reflected by reductions of 59% in the endpoints (*p* < 0.001) and 58% in the process length (*p* < 0.001) compared with the sham group. Additionally, the examination of the phagocytosis of Aβ deposits by activated microglia was performed. Confocal Z-stack series with high magnification were acquired and we found engulfing of Aβ deposits by few microglia in the sham group (Figure 9B-C). However, following the application of FUS/MB, internalization of Aβ deposits by microglia were extensive surrounding the depositions (Figure 9B-C), also as revealed by high-resolution 3D reconstruction compared with the Sham group (Movie S1 and S2).

### Proteomics changes in the hippocampus of 3×Tg-AD mice after FUS/MB treatment

#### Differentially expressed hippocampal proteins between 3×Tg-AD mice and WT mice

**Figure 10:**
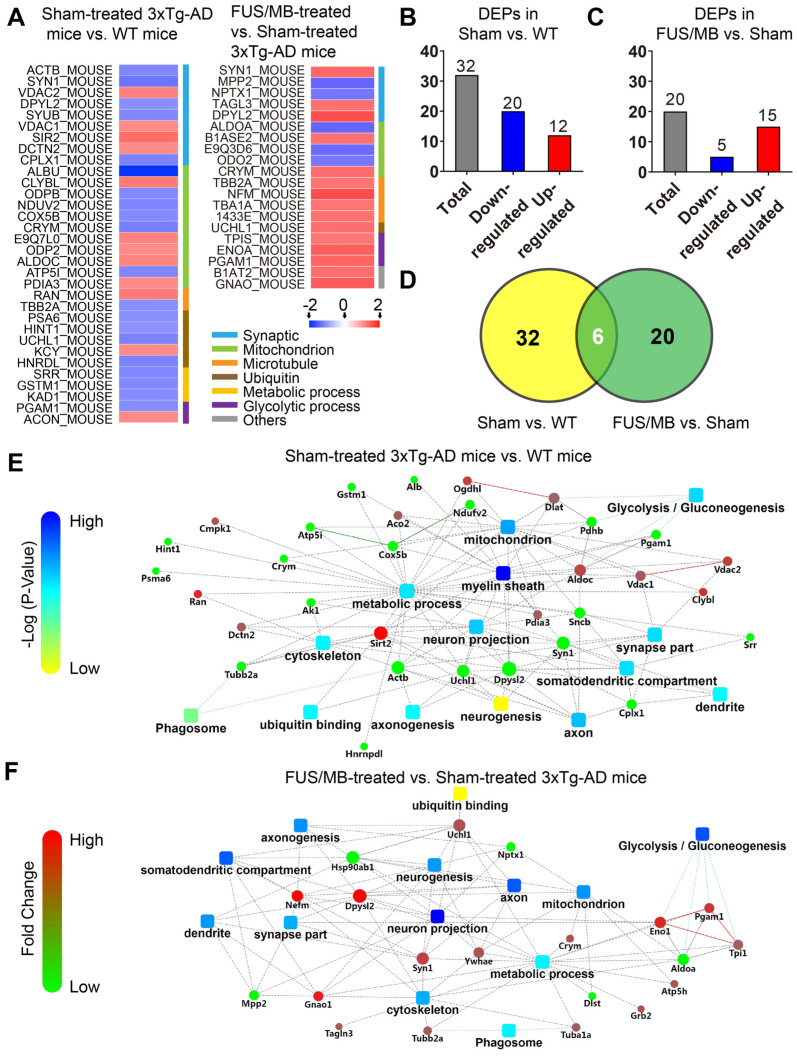
** Differentially expressed proteins (DEPs) and protein-protein interaction (PPI) networks in the hippocampus identified between the sham-treated 3×Tg-AD mice and WT mice (sham vs. WT), as well as the FUS/MB- and sham-treated 3×Tg-AD mice (FUS/MB vs. sham).** (**A**) Heat map showing DEPs in the two pairs of groups. Upregulation: red. Downregulation: blue. (**B-C**) The number of DEPs in the two pairs of groups. There were 32 differentially expressed proteins, with 12 proteins upregulated and 20 downregulated, in the sham group compared with the WT group (B). There were 20 DEPs, with 15 protein spots upregulated and five downregulated, in the FUS/MB group compared with the sham group (C). (**D**) In the DEPs of two pairs of groups, six proteins spots showed reversed expression levels. (**E**) The PPI network, indicating the interactions of DEPs with each other, in the sham vs. WT group. It mainly includes metabolic pathways, glycolysis, ubiquitin binding, neuronal parts, phagosome, etc. (**F**) The PPI network of the DEPs in the FUS/MB vs. sham group, showing close association with glycolysis, neuron projection, mitochondrial pathways, as well as metabolic process, phagosome, axon, neurogenesis parts and ubiquitin binding etc.

Proteomic analysis revealed that there were 32 proteins identified as differentially expressed proteins (DEPs) between the sham-treated 3×Tg-AD mice and WT mice (sham vs. WT, Table S1, Figure 10A). We carried out hierarchical heatmap clustering analysis in all hippocampal DEPs after they were normalized by the abundance of each protein in the WT group. Red represents upregulation and blue represents downregulation as shown in Figure 10A. These DEPs were mainly classified into synaptic, mitochondrion, ubiquitin, microtubule, metabolic and glycolytic process. Of these, 12 proteins were upregulated and 20 proteins were downregulated in the sham-treated 3×Tg-AD mice compared with the WT mice (Figure 10B).

Changes in the protein status are implicated in the regulation of biological functions. To understand the biological significance of the upregulated or downregulated proteins observed in the 3×Tg-AD mouse hippocampus, we performed gene ontology analysis utilizing the DAVID Classification System, in which proteins and their coding genes were classified on the basis of biological processes, molecular function and cellular component. As shown in Figure S7A, the biological processes of DEPs belonged to different classes, with most falling into the transport, metabolic process and tricarboxylic acid cycle categories. Molecular function analysis indicated that changes of the proteins were mainly connected with neuronal and metabolic processes. Furthermore, a large percentage of the proteins were associated with cellular components, including myelin sheaths, extracellular exosomes, mitochondria, cytoplasm, and axons.

To further understand the biological roles of DEPs and the signaling events they regulate, we performed PPI network analysis using the DAVID online database (Figure 10E). Analysis of PPI network indicated the interactions of DEPs with each other, including metabolic pathways, glycolysis, ubiquitin binding, neuronal parts, phagosome etc.

#### Differentially expressed hippocampal proteins between the FUS/MB-treated and sham-treated 3×Tg-AD mice

Twenty DEPs were identified in FUS/MB-treated mice as compared with sham-treated 3×Tg-AD mice (FUS/MB vs. sham, Table S2, Figure 10A). Among these, 15 proteins were upregulated in the FUS/MB group, including synaptic proteins (SYN1, DPYL2), microtubule proteins (TBA1A, TBB2A), mitochondrial proteins (CRYM, B1ASE2), glycolytic proteins (PGAM1, TIPS, ENOA) and an ubiquitin protein (UCHL1), etc. Moreover, five proteins were downregulated in the FUS/MB group, including mitochondrial proteins (ALDOA, E9Q3D6) and synaptic proteins (NPTX1) etc.

Biological processes in gene ontology analysis revealed that most of the DEPs fell into the glycolytic process, metabolic process, microtubule-based process and mitochondrial transport categories (Figure S7B). Molecular function analysis showed that protein kinase binding, cadherin binding, poly-A RNA binding, GTP binding and ubiquitin protein ligase binding were mainly involved (Figure S7B). Furthermore, many cellular components were associated, including myelin sheath, extracellular exosome, mitochondrion, cytoplasm, axon and neuron projections etc. PPI network showed close association with glycolysis, neuron projection, mitochondrial pathways, as well as metabolic process, phagosome, axon, neurogenesis parts and ubiquitin binding etc. (Figure 10F).

Interestingly, six proteins (SYN1, DPYL2, CRYM, PGAM1, UCHL1, TBB2A) were found to be commonly differentially expressed in the sham-treated 3×Tg-AD vs. The WT group and the FUS/MB-treated vs. sham-treated 3×Tg-AD group (Figure 10D). In the FUS/MB-treated group, these proteins showed reversed expression. Synaptic protein (SYN1, DPYL2), mitochondrial protein (CRYM), glycolytic protein (PGAM1), microtubule protein (TBB2A), and ubiquitin protein (UCHL1) were downregulated in the hippocampus of sham-treated 3×Tg-AD mice vs. WT mice, but upregulated in the FUS/MB group vs. the sham group. Proteins SYN1, PGAM1 and UCHL1 were also examined by western-blot analysis (Figure S7C). Quantification of the blots showed reversed expression of the proteins induced by FUS/MB treatment (Figure S7D).

These findings collectively suggested that the alterations of proteomic changes induced by FUS/MB treatment in the early-moderate stage of 3×Tg-AD mice might correlate with and contribute to improved neuronal function through various processes, including synaptic transmission and microtubule, ubiquitin, glycolytic and metabolic processes.

## Discussion

In this study, we investigated the effect of low-intensity FUS coupled with systemic administration of MBs on memory, pathology and proteomic changes in a triple-transgenic AD model, which displayed both progressive Aβ deposits and phosphorylated tau pathology with a similar spatial and temporal profile to that observed in human AD patients. The application of six-week FUS/MB treatment to the hippocampus of eight-month-old 3×Tg-AD mice led to clear improvement of their memory, as assessed by different hippocampus dependent cognitive tasks. In addition to the beneficial effects on behavioral tests, FUS/MB ameliorated Aβ deposits and mitigated tau pathology in the hippocampus. Further proteomic analysis revealed that various proteins were involved in several functions after FUS/MB treatment, including in synaptic transmission, microtubule-based processes, ubiquitinylation and metabolic processes.

Cumulative preclinical studies found evidence that repeated low-intensity FUS combined with MBs could induce safe BBB opening without neuron damage or hemorrhage when the therapeutic parameters were well controlled 38-40. The extent of BBB disruption could mainly be affected by the transducer frequency, ultrasound intensity, MB size and dosage 7, 41, 42. As for ultrasonic parameters, mechanical index (MI) has been reported to correlate well with the threshold (50% probability) for BBB disruption 41. The MI value, which is peak negative pressure amplitude estimated *in situ* divided by the square root of frequency, appeared to be constant at 0.46. In addition, MB size and dose can also have significant impacts on the outcome of BBB opening. Generally, larger MBs or higher dosages would increase the extent of BBB opening but could also induce a higher risk of damage to the brain given the same ultrasound parameters 42, 43. Therefore, heterogeneous parameters were used among studies with different experimental setups. The parameters utilized for the FUS/MB procedure in this study were originally derived from our previous study 43. After accounting for 18.1% mouse skull attenuation, as reported in Choi's study 7, the MI value in this study was estimated to be 0.53. Using a MB dosage of 0.2 µL/g (body weight) (concentration: 1-3×10^9^/mL), the number of MBs injected into a mouse's body was estimated to range from 2×10^5^/g to 6×10^5^/g. Under this set of parameters, we found stable BBB opening in 3×Tg-AD mice, and no gross tissue damage was revealed by histological assessment. The BBB would start to restore within 24 hours after the disruption by FUS/MB. We found a transient and slight weight decrease induced by the stress from repeated treatments. The body weights of the sham group and FUS/MB group mice decreased by 3% and 2.3%, respectively, after one-week treatment and then gradually recovered, while only FUS did not exert a deleterious effect on the animal's condition (Figure S8). Thus, the repeated ultrasound treatments we applied were tolerated by the 3×Tg-AD mice at this age. Recently, a phase I clinical trial conducted by Lipsman et al. on five AD patients demonstrated that safe, reversible and repeated opening of BBB could be achieved using MRgFUS with circulating MBs at an average exposure power of 4.6 W with Definity (dose: 4 μL/kg), making this modality a promising strategy for clinical AD treatment 12.

Extensive evidence has shown that BBB integrity is compromised in AD brains. Extravasation of blood-derived macromolecules (fibrin, IgG, albumin, thrombin, plasmin) or intravenously administrated tracers (Evans blue, contrast agents) has been shown to deposit perivascularly in various AD models or patients 17-20, 44. In particular, the persistent deposition of fibrin accelerates neurovascular damage and drives a chronic inflammatory state, further promoting neuronal degeneration 19, 44-46. Although extensively reported in many other AD models, we found no Evans blue extravasation or fibrinogen leakage to the brain interstitium in the untreated hemisphere of eight-month-old 3×Tg-AD mice in the present study. The time course study of BBB opening after FUS/MB indicated its closure within 24 h, which is consistent with other studies 47. Granted, immediately after the BBB was opened by FUS/MB, fibrinogen extravasation was detected. However, at 6 h after the treatment, most of the fibrinogen had been cleared, as indicated by the loss of a positive immunofluorescence signal. Unlike the toxicity induced by persistent accumulation, Montagne et al. found that the administration of transient fibrinogen or fibrin fibrils to oligodendrocytes within 6 or 12 h did not lead to cell death *in vitro,* but did activate autophagy, a cell-degrading process associated with metabolic stress 22. Although not included in this study, comprehensive investigation into the distress elicited by FUS/MB-induced transient BBB opening is warranted for further development of this technique in the therapeutic application. In addition, low-density lipoprotein receptor-related protein 1 (LRP1), has been shown to be an important mediator for the transvaslular clearance of brain-derived Aβ. Diminishing of LRP1 expression at the BBB has been shown in transgenic AD models and led to brain accumulation of Aβ 17. Through genetic deletion of LRP1 in the brain endothelium, Storck et al. found reduced plasma Aβ levels in 5xFAD mice 48. Thus, restoring BBB through regulation of neurovascular pathways, such as targeting at LRP1 and phosphatidylinositol-binding clathrin assembly protein (PICALM) etc., has gained much attention for a potential therapeutic strategy for AD therapy 21. The effects of FUS/MB on these pathways have been unknown yet and need to be further investigated.

Various transgenic AD models were used in the studies of exploring the effect of FUS/MB on AD 11, 13, 15, 25. Some models develop amyloid pathology such as TgCRND8 mice and APP23 mice or tau pathology such as P301L mice. However, to the best of our knowledge, 3×Tg-AD mice, which display both amyloid and tau pathology, have not previously been formally utilized to explore the therapeutic effect of FUS/MB, including pathology and behaviors. This model developed intraneuronal Aβ and synaptic dysfunction preceding extracellular plaque formation and tangles in mice as young as three months 27, 28. In our study, eight-month-old female 3×Tg-AD mice were used because both Aβ and phosphorylated tau pathology were observed at this age and no diminished traits have been detected in female mice through generations. Specifically, intraneuronal Aβ in the cortex, hippocampus, and amygdala was prominent. However, large areas of amyloid plaques had not yet developed in the cortex or hippocampal formation. Despite this, extracellular diffuse Aβ deposits were apparent in the SR layer of the hippocampus. As for tau pathology of 3×Tg-AD mice, AT180-immunoreative neurons were reported in the hippocampus as early as 6-month old and robust positivity was apparent at nine months of age 36, 49. Sparse AT8-positive neurons could be observed in the hippocampus at six months of age 36, 50. As the mice aged, neurons immunoreactive to both the two antibodies could be detected. We also found that AT180-positive neurons displayed greater numbers and wider spatial distributions than AT8-positive neurons in the 3×Tg-AD mice, in line with other reports 36. When the mice were in the middle and late stages of the disease progression, the AT8 signal intensified 36, 49. According to the existing literature depicting temporal and spatial Aβ and tau pathology of 3×Tg-AD mice, we speculated that the mice we used were in the early-moderate stage of disease progression 36, 49-51. The occurrence of both Aβ and tau pathology in 3×Tg-AD mice allowed us to assess whether FUS/MB could exert any beneficial influences on the two hallmark lesions of AD.

Intriguingly, a handful of recent studies demonstrated that even without any therapeutic agents, FUS/MB alone could alleviate cognitive impairment in transgenic AD mice 14, 16, 26. After bilateral sonication of the hippocampus once per week for one month, the time that TgCRND8 mice spent in exploring the novel arm of a Y maze showed 99% increase compared with untreated mice, showing restoration of spatial working memory to the wild-type level 14. In our study, the Y-maze task was also utilized to assess the short-term working memory of the animals. Data of the number of entries, distance and duration in the novel arm showed similar findings with them. Leinenga et al. conducted SUS treatment with circulating MBs in the entire brains of APP23 mice 16. When tested by an active place avoidance task, SUS-treated mice received fewer shocks in the retesting session after a seven-week treatment period, showing improved performance of reversal learning and long-term memory. Using K369I tau transgenic mice with an early-onset tau phenotype, improved motor and memory functions were induced by repeated SUS/MB with a long treatment session of 15 weeks 26. In the present study, as a prevalent and reliable behavioral test correlated with hippocampal synaptic plasticity, the Morris water maze was chosen to assess spatial learning and long-term memory in the FUS/MB-treated AD mice. Spatial learning was obtained across repeated trials using distal cues to navigate the animals to the hidden platform. To evaluate long-term memory, the reference memory was assessed seven days after the last learning trial, by preference for the platform area when the platform was removed. The earliest cognitive impairment was reported to emerge in the 3×Tg-AD mice at four months of age tested by Morris water maze 35. Both the short-term and long-term deficits developed subsequently at six months of age 35. Likewise, in our study, the escape latency of the sham group in the training days and multiple indices of memory in the probe trial revealed significant memory impairment in the 3×Tg-AD mice we used. Conspicuously, FUS/MB-treated mice displayed less escape latency to find the hidden platform after the 4th day in the training session, as well as noticeable reduction in probe time during the probe trial compared with sham or FUS group. Additionally, the number of crossings, time and distance in the platform quadrant increased exceptionally. In line with the findings of the above studies of other groups, our study indicated that FUS/MB treatment, in the absence of therapeutic agents, substantially improved the learning and memory of AD transgenic mice despite of the disparities in the animal models.

Furthermore, emerging evidence demonstrated that repeated FUS/MB treatment alone could result in significant reduction of amyloid plaques in AD mouse models 14, 16. Using seven-month-old TgCRDN8 AD mice with abundant extracellular amyloid deposits and dense-cored plaques, Burgess et al. found that the mean plaque size and total plaque number could be reduced by 27% and 29%, respectively, compared with the non-Tg littermates after weekly FUS/MB exposure to the hippocampus for one month 14. At an age of 12 to 13 months, APP23 transgenic mice with pronounced mature amyloid plaques mainly in the cortex showed remarkable Aβ plaque clearance after four weeks of repeated SUS/MB treatment 16. As a parallel to their findings, our results showed that extracellular Aβ deposits in the hippocampi of 3×Tg-AD mice were reduced after repeated FUS/MB treatments. It should be noted that the intraneuronal Aβ pathology was also ameliorated in both hippocampi and amygdala parenchyma for the FUS/MB-treated mice. Many studies have found that intraneuronal Aβ is cytotoxic to neurons and correlated with synaptic dysfunction or neuron loss 52, 53. The accumulation of intraneuronal Aβ was the first pathological manifestation and triggered the cognitive deficits in 3×Tg-AD mice 35. Our study also showed that distinct memory impairment in the 3×Tg-AD mice has already developed preceding the accumulation of mature plaques in the extracellular cerebral regions. It was shown that clearance of intraneuronal Aβ by anti-Aβ antibody injections reversed the early cognitive impairment which retrogressed when the Aβ pathology reappeared one month after the cession of the immunotherapy 35. Along with the findings of our behavioral tests, it is plausible that the cognitive memory improvement in FUS/MB-treated mice is likely to correlate with the alleviation of intraneuronal Aβ accumulation. One of the limitations in the present study is that soluble or insoluble Aβ levels were not identified, nor were oligomeric types. The antibody we used can recognize several types of Aβ peptide, such as Aβ 1-40, Aβ 1-42, and Aβ 1-16/17. Nevertheless, Leinenga et al. reported a twofold reduction in Aβ42, along with monomer and trimer decreases by fivefold and twofold, respectively, in SUS-treated APP23 mice compared with sham-treated mice 16. Given the disparities between the models of other studies and our model, this needs to be further investigated.

As another hallmark of AD pathogenesis, the aberrant phosphorylated tau is believed to closely correlate with neuronal degeneration and cognitive dysfunction, making tau an attractive target in AD therapy 54, 55. Very few studies have explored the effect of FUS/MB on tau pathology using transgenic mice without therapeutic agents 25, 26. Using a genotype of the tau mouse model rTg4510 line, the efficacy of unilateral FUS/MB on early-stage tau pathology was investigated in the hippocampus 25. Reduction of phosphorylated tau from the hippocampal formation processes especially the pyramidal CA1 neurons was observed in the sonicated hemisphere, which was closely related to the increased activation of microglia engulfing phosphorylated tau 25. Interestingly, unilateral ultrasound application induced p-tau reduction in bilateral hippocampus of the rTg4510 mouse model. However, in our study, this bilateral effect was not observed indicated by no significant difference of p-tau in the contralateral hemispheres between FUS/MB-treated mice and sham-treated mice (Figure S6). Another concurrent study revealed a significant reduction of tau with improvements in impaired motor and memory function induced by weekly SUS/MB treatment for up to 15 weeks in K369I tau transgenic mice 26. Notably, this study suggested a new tau-clearing mechanism correlated with autophagy. The analysis of AT8- and AT180-immunoreative neurons in our study showed that the early tau phosphorylation in 3×Tg-AD mice was effectively mitigated in the sonicated hippocampus. Oddo et al. provided compelling evidence that single intrahippocampal injection of anti-Aβ antibodies led to a distinct reduction of the early but not the late forms of phosphorylated human tau in 12-month-old 3×Tg-AD mice, following the clearance of intracellular and extracellular Aβ 56. In contrast, hyperphosphorylated tau aggregates were resistant to the anti-Aβ immunotherapy as determined by HT7 immunostaining. In addition, a growing body of pre-clinical studies has shown that the reduction in Aβ contributes to alleviation of phosphorylated tau, and subsequently improved cognitive dysfunction in 3×Tg-AD mice 57, 58. However, the possible mechanisms of FUS/MB treatment in tau pathology have been far from fully understood. As an initial finding of amelioration in tau pathology triggered by FUS/MB treatment in 3×Tg-AD mice, further evidence supporting the correlation with the Aβ reduction should be profoundly investigated.

Through comparisons of the pathology between contralaterally non-sonicated and sonicated hemispheres, our study found remarkable attenuation of Aβ deposits and p-tau in the sonicated hemisphere. Additionally, we saw clear behavioral benefits of the treatment even though it was applied only unilaterally. We also found improved neuron axonal health in the hippocampus through immunostaining against phosphorylated neurofilament proteins in axons but without benefits in NeuN-positive neurons emerging from the repeated FUS/MB treatments. However, it was reported that FUS/MB induced BBB opening could stimulate a response of neurogenesis in the hippocampus in non-transgenic mice and AD animal models 14, 59-61. Scarcelli et al. demonstrated that FUS/MB treatment (10 ms bursts at 1-Hz PRF, 0.96 MPa, 120 seconds) was able to increase neurogenesis by more than twofold in the dentate gyrus (DG) subregion of non-transgenic mice 59, and Mooney et al. demonstrated that the neurogenesis was associated with an increase in BBB permeability 60. Correlating with spatial memory improvement, Burgess et al. found a 250% increase in the number of newborn doublecortin (DCX)-positive neurons in the DG subregion after four weeks of MRgFUS/MB treatments. Although the underlying mechanisms are still unknown, the activation of the cellular molecular signaling cascade may be involved. It has also been recently reported that a single FUS/MB treatment (10 ms bursts at 1-Hz PRF, 0.25 MPa, 120 s) elicited a 35% increase in brain-derived neurotrophic factor expression in the hippocampus in a rat model with cholinergic degeneration 61. Furthermore, Hatch et al. proved that SUS treatment could prevent loss of dendritic structure as the wild-type mice aged 62. Taken together, these findings suggest that using FUS/MB-mediated BBB opening to improve neuronal health is a potential therapeutic strategy for AD treatment.

Based on these beneficial effects of FUS/MB treatment on 3×Tg-AD mice, we sought to discover the changes of proteomic profile related with the amelioration of pathology and improvements in cognitive performance of the AD animals. In the BBB opening experiment, Evans blue indicated that FUS alone could not disrupt BBB function, using the same sonication parameters as the FUS/MB group. Moreover, the following behavioral and histological analysis showed no improvement in the FUS group. We therefore decided to focus on the comparison between FUS/MB-treated and sham-treated AD animals, trying to discover some differentiated expressed proteins. Mass spectrometry combined with 2D-DIGE indicated that between FUS/MB- and sham-treated mice, differentially expressed proteins were found to associate with synapse, microtubule, mitochondrion, metabolic process and ubiquitin. Common with DEPs in sham vs. WT mice, the expression of six proteins (SYN1, DPYL2, CRYM, PGAM1, UCHL1, TBB2A) was reversed. SYN1 is a major neuron specific phosphoprotein localized in the small synaptic vesicles, which performs important functions in synapse formation, vesicle trafficking, and neurotransmitter release and was found to be decreased in the AD brain 63. DPYL2 is normally concentrated within the neurites and synapses, and is mostly expressed in the hippocampus, olfactory bulb and cerebellum 64. It plays important roles in regulating neurite structure, vesicle trafficking and ion channel function 65, and was reported to be downregulated and oxidatively modified in AD brains 66 and 3×Tg-AD mice 67. PGAM1 is an enzyme of the glycolysis pathway involved in mitochondrial-mediated processes and was reported to be downregulated in 3×Tg-AD mice 68. The upregulation of PGAM1 could promote cell proliferation and neuroblast differentiation in the dentate gyrus 69 and provide protective effects against Aβ-induced cell death 68. TBB2A is a subtype of β-tubulin, one of the polymerization components of dynamic microtubules and was found to be downregulated in 3×Tg-AD mice 70. As one of the most abundant proteins in the brain, UCHL1 is essential for normal synaptic and cognitive memory function 71; it degrades misfolded proteins and recycles ubiquitin molecules under stress conditions 72. UCHL1 was found to be downregulated in 3×Tg-AD mice 67, 70. A recent study revealed that transduction of UCHL1 protein restored Aβ-deteriorated long-term potentiation and synaptic plasticity, leading to improved memory in APP/PS1 mice 73. It has been reported that intraneuronal Aβ can be degraded by ubiquitin-proteosome pathway 74, which has also been recognized to relate with the degradation of phosphorylated tau 75. Interestingly, reduced tau hyperphosphorylation was observed in tau transgenic mice after SUS treatment 11 and increased protein ubiquitinylation was found in neurons 76, suggesting ubiquitin as a possible pathway to ameliorate tau pathology. However, contradiction was indicated in Pandit et al.'s study as they found no increase in ubiquitinated tau following repeated SUS treatments in K369I mice 26, which may derive from different animal models used. Instead, they found significant differences of markers in autophagy pathways in the tau transgenic mice after SUS treatments and suggested a tau clearance mechanism involving autophagosomes. Nonetheless, our proteomic analysis revealed that AD-related pathways such as glycolysis, metabolic process and ubiquitin binding were associated with FUS/MB treatment. Moreover, positive effect on neuron-related proteins has been elicited by this regime, including neuron projection, cytoskeleton, dendritic etc., seemingly co-relating with improved axonal health found by immunohistological assessment and attenuation of the pathology. Although our proteomic analysis was quite preliminary, the alterations of the proteins we identified may provide potential hints to further investigate the downstream events induced by FUS/MB treatment on AD.

Cerebral amyloid angiopathy (CAA), which involves deposition of Aβ peptides in cerebral vessels, is one of the most common vascular pathologies present in AD patients 77 and transgenic AD models 78. CAA has been related to a high-risk factor for the occurrence of cerebral microbleeds and intracerebral hemorrhages 77. Given the high overlap between CAA and AD, concerns about the consequences of FUS/MB-induced BBB opening on CAA remains an important issue. Using aged APP23 mice (two years old), it was found that SUS treatment with MBs neither augmented microbleeds nor affected CAA pathology, compared with the sham group 79. In addition, the *in vivo* observation of BBB opening by two-photon microscopy revealed different leakage kinetics between transgenic AD mice and non-transgenic mice 80. Studies reported considerable levels of APP/Aβ deposition in the cerebral microvessels of transgenic 3×Tg-AD mice, found by immunohistochemical analysis of brain sections 81, 82. Future studies must to investigate the effect of FUS/MB treatment with this study's parameters on CAA in 3×Tg-AD mice, as this issue is of major importance when considering the clinical applicaiton of this technique.

## Conclusions

In summary, our findings indicate that repeated low-intensity FUS/MB led to evident improvement of memory impairments, concurrent with amelioraton of both Aβ and tau pathology, in a triple transgenic AD mouse model in an early-moderate stage of pathology. FUS/MB treatment improved hippocampal neuron axonal health in 3×Tg-AD mice and activated the phagocytosis of Aβ deposits by microglia. Additionally, the alteration of hippocampal proteomic changes revealed that various proteins were involved after FUS/MB treatment, including synaptic, microtubule, ubiquitin and metabolic process. The results in this study reinforce the positive therapeutic effects on AD models induced by FUS/MB-mediated BBB opening, further supporting the potential of this treatment regime in clinical applications.

## Supplementary Material

Supplementary figures and tables.

Supplementary movie S1.

Supplementary movie S2.

## Abbreviations

AD: Alzheimer's disease
BBB: blood-brain barrier
FUS: Focused ultrasound
MBs: Microbubbles
MRgFUS: magnetic resonance-guided focused ultrasound
Aβ: Amyloid-β
SUS: scanning ultrasound
3×Tg-AD: triple transgenic AD
WT: wild type
2D-DIGE: two-dimensional fluorescence difference gel electrophoresis
H&E: hematoxylin and eosin
DAVID: the Database for Annotation, Visualization and Integrated Discovery Resource
EB: Evans blue
SR: stratum radiatum
DEPs: differentially expressed proteins
