# Supplementary material for: Ultrasound with microbubbles improves memory, ameliorates pathology and modulates hippocampal proteomic changes in a triple transgenic mouse model of Alzheimer's disease
Source: Theranostics. 2020 Sep 26;10(25):11794–819. doi: 10.7150/thno.44152 (PMC7546002; doi:10.7150/thno.44152)
Supplement: Supplementary file 1 — Supplementary figures and tables. [file thnov10p11794s1.pdf]

Supplemental figures and tables

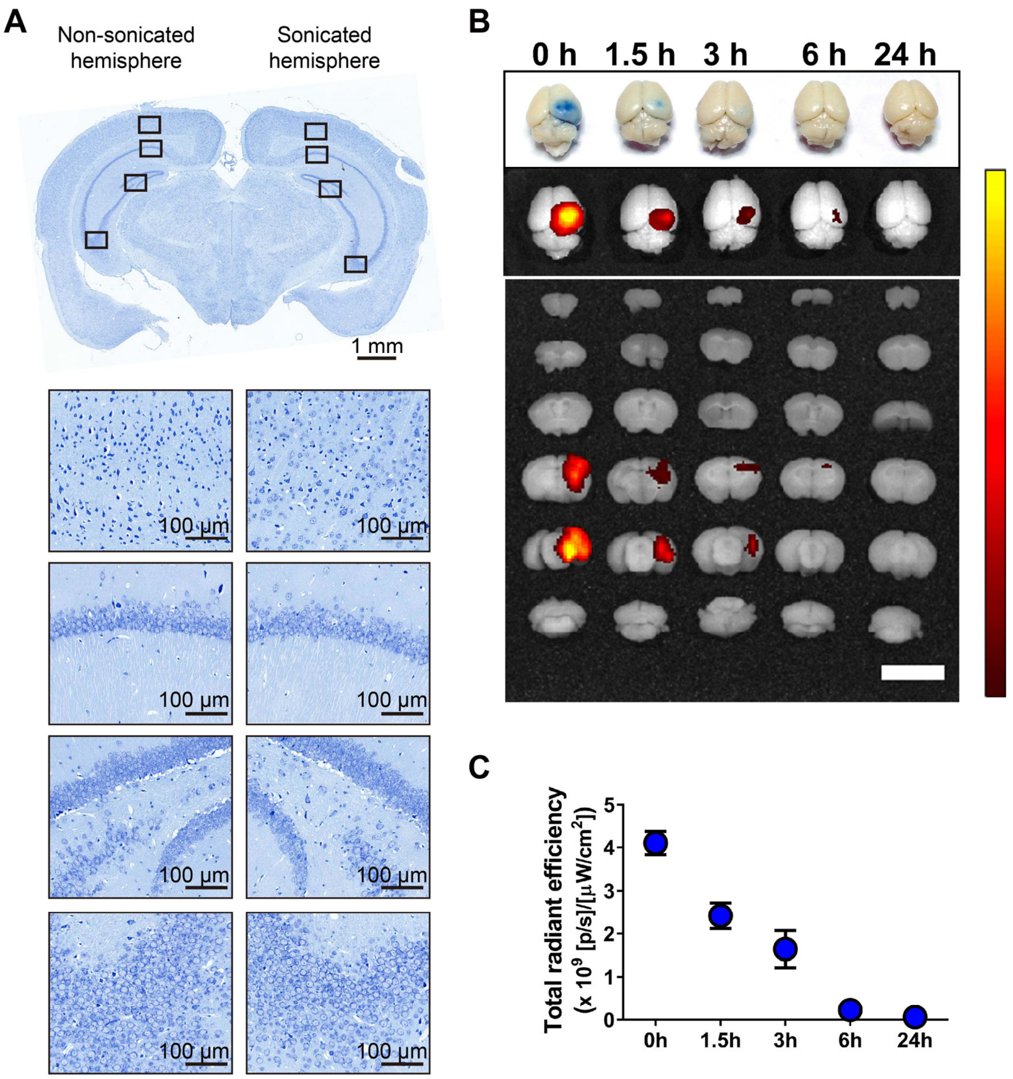

Figure S1. (A) Representative images of Nissl staining of brain sections in 3×Tg-AD mice after BBB opening induced by a one-time FUS/MB treatment. Upper left: the non-sonicated side. Upper right: the sonicated side. Lower left and right: images with high magnification in the black solid boxes. Upper scale bar: 1 mm, scale bars in the lower subfigures: 100 μm. No abnormalities in neuron integrity were discernible compared with the contralateral region. (B) The time course of BBB opening by FUS/MB treatment. Evans blue (EB) was injected at the time points of 0 h, 1.5 h, 3 h, 6 h and 24 h after the treatment (n = 3–5 for each group), circulating for another two hours. Scale bar: 1 cm. (C) Total radiant efficiency of the EB extravasated into the brain tissue showed BBB closure in 24 hours after FUS/MB treatment.

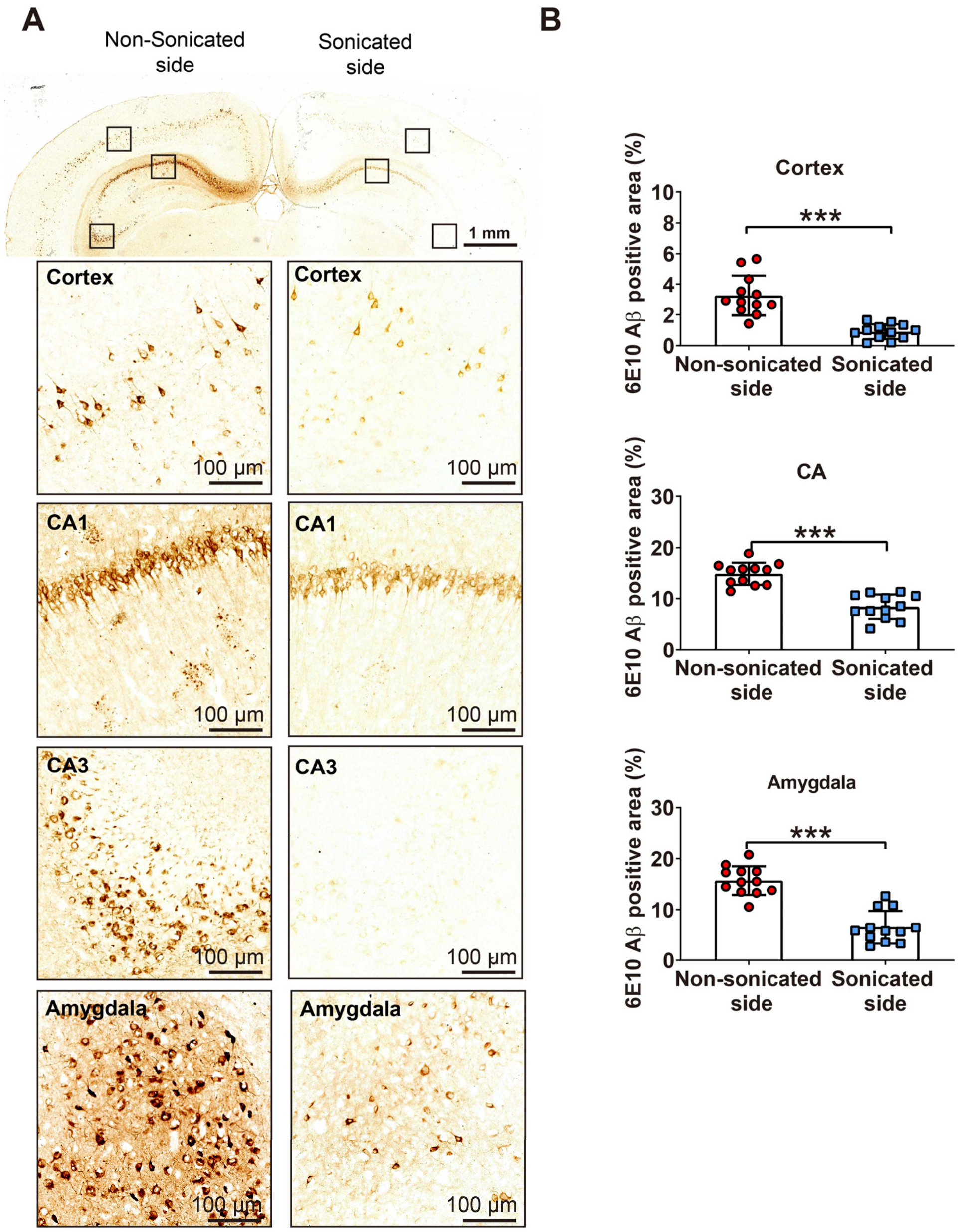

Figure S2. Aβ pathology in the contralaterally non-sonicated (left) and sonicated (right) hemispheres of the 3×Tg-AD mice treated by FUS/MB for 6 weeks (twice per week). The brain sections were immunostained with anti-Aβ antibody 6E10. Scanning of the whole brain section was performed. (A) Representative immunohistochemical images from the medial coronal brain section. Remarkable distinction of 6E10 immunoreactivity between the non-sonicated and sonicated hemispheres could be observed in the cortex, CA1 and CA3 regions, and amygdala. Upper scale bar: 1 mm, scale bars in the lower subfigures: 100 μm. (B) Quantitative analysis of the 6E10-positive areas in the cortex, CA region, and amygdala of the two hemispheres. The 6E10-positive areas in the cortex, CA region, and amygdala of the sonicated side from medial brain sections reduced by orders of 72%, 43%, and 58% compared with the contralaterally non-sonicated side. Paired *t*-tests were used. ***: $p < 0.001$.

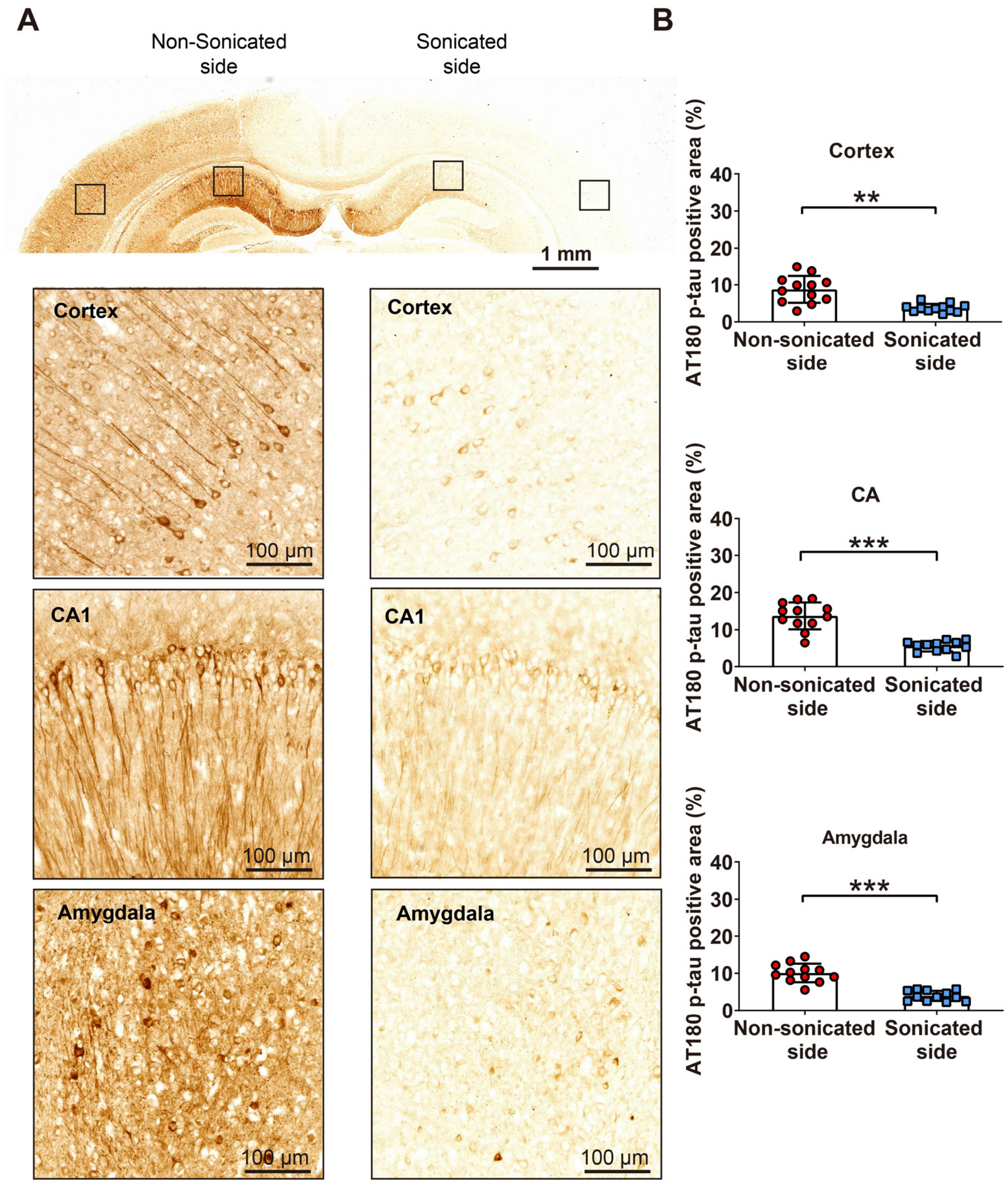

Figure S3. Phosphorylated tau stained with AT180 in the contralaterally non-sonicated (left) and sonicated (right) hemispheres of the 3×Tg-AD mice treated by FUS/MB for 6 weeks (twice per week). Scanning of the whole brain section was performed. (A) Representative immunohistochemical images from the medial coronal brain section. Substantial differences of AT180 immunoreactivity between the non-sonicated and sonicated hemispheres could be observed in the cortex, CA1 subregion, and amygdala. Upper scale bar: 1 mm, scale bars in the lower subfigures: 100 μm. (B) Quantitative analysis of the AT180-positive areas in the cortex, CA subregion, and amygdala of the two hemispheres. The AT180-positive areas in the cortex, CA subregion, and amygdala of the sonicated side from medial brain sections reduced by orders of 57%, 60%, and 61% compared with the contralaterally non-sonicated side. Paired *t*-tests were used. **: $p < 0.01$, ***: $p < 0.001$.

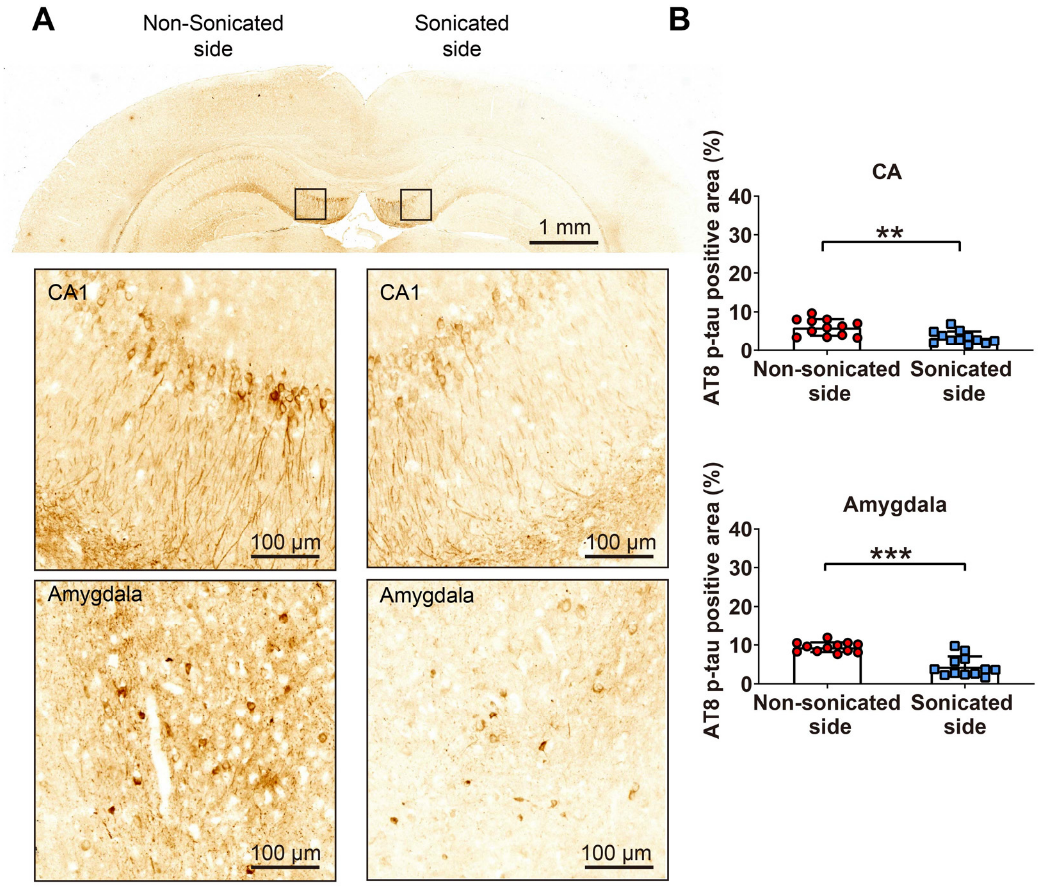

Figure S4. Phosphorylated tau stained with AT8 in the contralaterally non-sonicated (left) and sonicated (right) hemispheres of the 3×Tg-AD mice treated by FUS/MB for 6 weeks (twice per week). Scanning of the whole brain section was performed. (A) Representative immunohistochemical images from the medial coronal brain section. Substantial differences of AT8 immunoreactivity between the non-sonicated and sonicated hemispheres could be observed in the CA1 subregion and amygdala. Upper scale bar: 1 mm, scale bars in the lower subfigures: 100 µm. (B) Quantitative analysis of the AT8-positive areas in the CA region and amygdala of the two hemispheres. The AT8-positive areas in the CA and amygdala of the sonicated side from medial brain sections reduced by orders of 44% and 53% compared with the contralaterally non-sonicated side. Paired *t*-tests were used. **: $p < 0.01$, ***: $p < 0.001$.

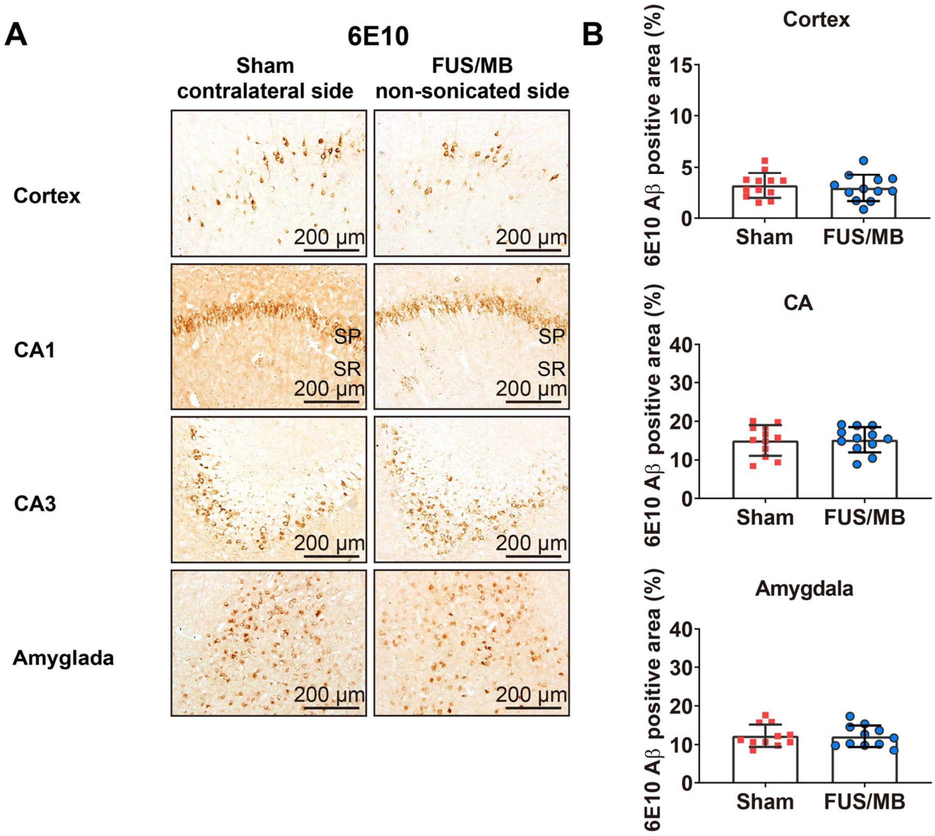

Figure S5. Aβ pathology stained with 6E10 in the non-sonicated hemisphere of the FUS/MB-treated 3×Tg-AD mice and the contralateral side of sham-treated mice. (A) Representative immunohistochemical images in the cortex, CA1 and CA3 subregions, and the amygdala of the non-sonicated hemisphere in the FUS/MB group and the contralateral side in the sham group. (B) Quantitative analysis of the 6E10-positive areas in the CA subregion, and amygdala of the two hemispheres. No substantial differences of 6E10 immunoreactivity between the non-sonicated and sonicated hemispheres could be observed in the contralateral hemispheres could be observed between the FUS/MB and sham group. Scale bar: 200 μm.

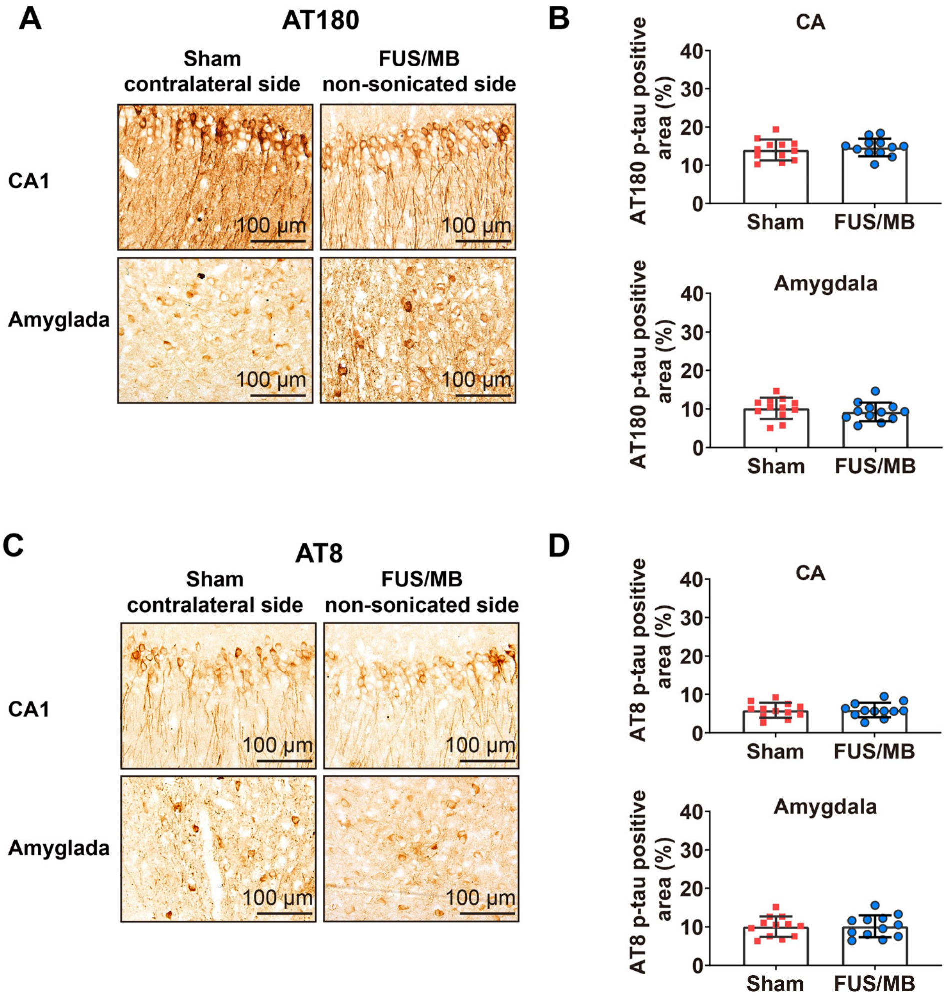

Figure S6. Phosphorylated tau stained with AT180 and AT8 in the non-sonicated hemisphere of the FUS/MB-treated 3×Tg-AD mice and the contralateral side of sham-treated mice. (A) Representative immunohistochemical images against AT180 in the CA subregion and amygdala of the non-sonicated hemisphere in the FUS/MB group and the contralateral side in the sham group. (B) Quantitative analysis of the AT180-positive areas in the CA subregion, and amygdala of the two hemispheres. (C) Representative immunohistochemical images against AT8 in the CA subregion and amygdala of the non-sonicated hemisphere in the FUS/MB group and the contralateral side in the sham group. (D) Quantitative analysis of the AT8-positive areas in the CA subregion, and amygdala of the two hemispheres. No substantial differences of AT180 and AT8 immunoreactivity in the contralateral hemispheres could be observed between the FUS/MB and sham group. Scale bar: 100 μm.

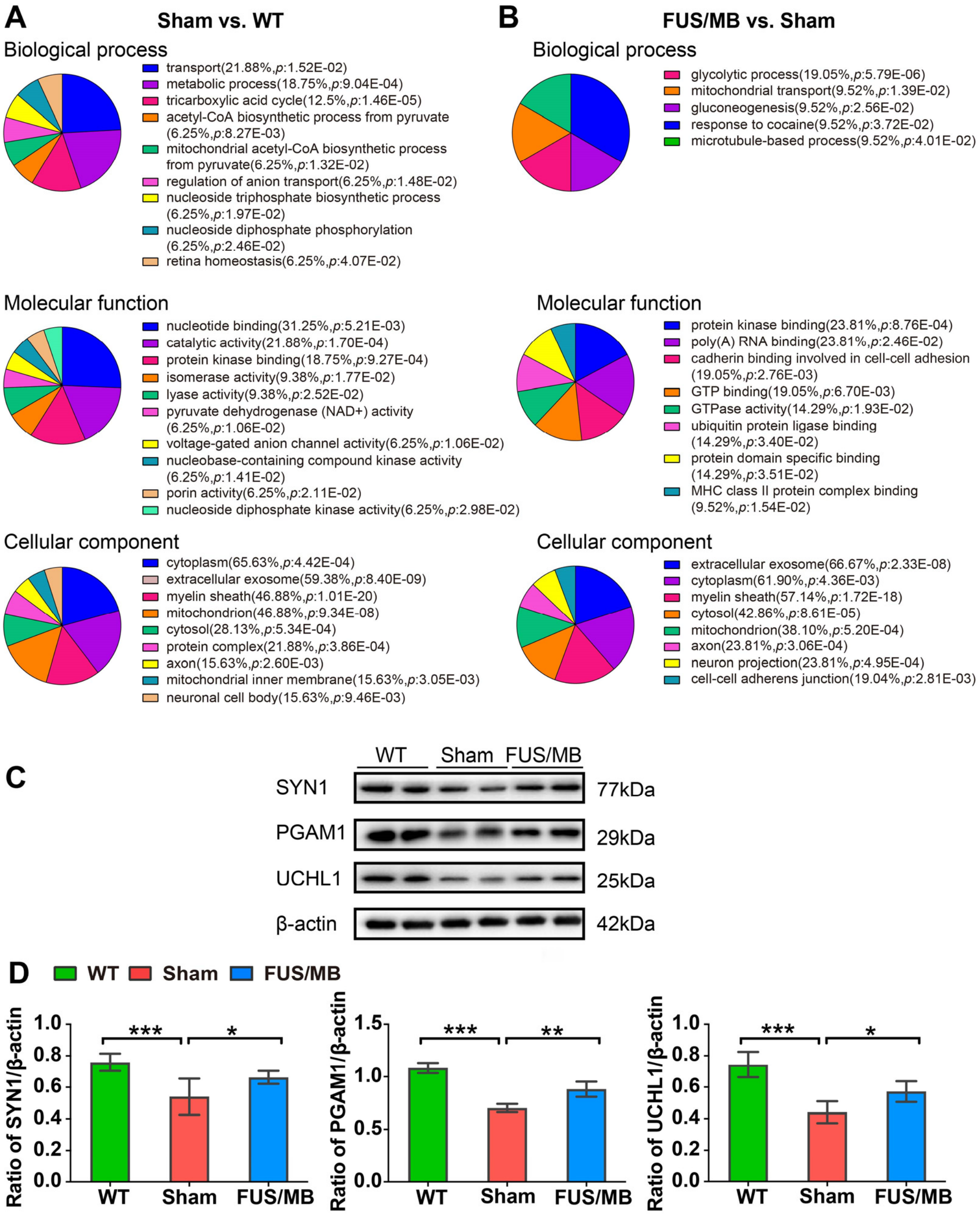

Figure S7 (A-B) DAVID Gene ontology enrichment analysis of the differentially expressed proteins in hippocampus between the sham-treated 3×Tg-AD mice and WT mice (sham vs. WT), as well as the FUS/MB- and sham-treated 3×Tg-AD mice (FUS/MB vs. sham). Gene ontology terms ($p < 0.05$) are included in biological processes, molecular functions, and cellular components. (C) Representative image of western-blot verification. Proteins SYN1, PGAM1 and UCHL1 were verified by western-blot analysis. β-actin was used as a loading control. The western-blot analysis was performed in triplicate. (D) Quantification of the blots showed reversed expression of the proteins induced by FUS/MB treatment. *: $p < 0.05$, **: $p < 0.01$, ***: $p < 0.001$.

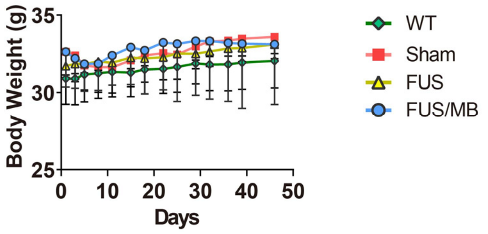

Figure S8. The body weights of the 3×Tg-AD mice receiving sham, FUS and FUS/MB treatment, as well as the WT mice, were monitored throughout the treatment period. A transient and slight weight decrease was induced by the stress from repeated treatments at the initial week. The body weights of the sham group and FUS/MB group mice decreased by 3% and 2.3%, respectively, after one-week treatment and then gradually recovered, while only FUS did not exert a deleterious effect on the animal’s condition. Thus, the repeated ultrasound treatments we applied were tolerated by the 3×Tg-AD mice at eight months of age.

Table S1. Differentially expressed proteins between the sham-treated 3×Tg-AD mice and WT mice.

No	Protein Name	Accession No.	Gene name	Mascot Score	Ratio	p-Value	Classification
**1**	Actin, cytoplasmic 1	ACTB_MOUSE	P60710	259	-1.14	0.00044	synaptic proteins
**2**	Serum albumin	ALBU_MOUSE	P07724	283	-1.97	0.00087	mitochondrion
**3**	Synapsin-1	SYN1_MOUSE	O88935	139	-1.31	0.00092	synaptic proteins
**4**	Citrate lyase subunit beta-like protein ,mitochondrial	CLYBL_MOUSE	Q8R4N0	58	1.21	0.0028	mitochondrion
**5**	Pyruvate dehydrogenase E1 component subunit beta	ODPB_MOUSE	Q9D051	345	-1.11	0.0039	mitochondrion
**6**	Proteasome subunit alpha type-6	PSA6_MOUSE	Q9QUM9	267	-1.08	0.004	ubiquitin
**7**	Serine racemase	SRR_MOUSE	Q9QZX7	271	-1.14	0.0047	metabolic process
**8**	NADH dehydrogenase [ubiquinone] flavoprotein 2	NDUV2_MOUSE	Q9D6J6	342	-1.16	0.0071	mitochondrion
**9**	Glutathione S-transferase Mu 1	GSTM1_MOUSE	P10649	347	-1.13	0.0073	metabolic process
**10**	GTP-binding nuclear protein Ran	RAN_MOUSE	P62827	89	1.21	0.0083	microtubule process
**11**	Cytochrome c oxidase subunit 5B	COX5B_MOUSE	P19536	321	-1.11	0.011	mitochondrion
**12**	Voltage-dependent anion-selective channel protein 2	VDAC2_MOUSE	Q60930	182	1.15	0.012	synaptic proteins
**13**	Histidine triad nucleotide-binding protein 1	HINT1_MOUSE	P70349	269	-1.06	0.014	ubiquitin
**14**	Thiomorpholine-carboxylate dehydrogenase	CRYM_MOUSE	O54983	353	-1.21	0.016	mitochondrion
**15**	Ubiquitin carboxyl-terminal hydrolase isozyme L1	UCHL1_MOUSE	Q9R0P9	222	-1.18	0.016	ubiquitin
**16**	Dihydropyrimidinase-related protein 2	DPYL2_MOUSE	O08553	538	-1.07	0.016	synaptic proteins
**17**	Beta-synuclein	SYUB_MOUSE	Q91ZZ3	123	-1.13	0.0019	synaptic proteins
**18**	Adenylate kinase isoenzyme 1	KAD1_MOUSE	Q9R0Y5	266	-1.1	0.021	metabolic process
**19**	Protein Ogdhl	E9Q7L0_MOUSE	E9Q7L0	111	1.15	0.027	mitochondrion
**20**	Tubulin beta-2A chain	TBB2A_MOUSE	Q7TMM9	373	-1.08	0.029	microtubule process
**21**	Voltage-dependent anion-selective channel protein 1	VDAC1_MOUSE	Q60932	355	1.09	0.029	synaptic proteins
**22**	Dihydrolipoyllysine-residue acetyltransferase component of pyruvate dehydrogenase complex	ODP2_MOUSE	Q8BMF4	359	1.07	0.03	mitochondrion
**23**	Fructose-bisphosphate aldolase C	ALDOC_MOUSE	P05063	123	1.13	0.032	mitochondrion
**24**	NAD-dependent protein deacetylase sirtuin-2	SIR2_MOUSE	Q8VDQ8	82	1.33	0.038	synaptic proteins

**25**	Phosphoglycerate mutase 1	PGAM1_MOUSE	Q9DBJ1	461	-1.09	0.041	glycolytic process
**26**	UMP-CMP kinase	KCY_MOUSE	Q9DBP5	278	1.09	0.042	ubiquitin
**27**	ATP synthase subunit e, mitochondrial	ATP5I_MOUSE	Q06185	251	-1.14	0.042	mitochondrion
**28**	Dynactin subunit 2	DCTN2_MOUSE	Q99KJ8	439	1.09	0.043	synaptic proteins
**29**	Heterogeneous nuclear ribonucleoprotein D-like	HNRDL_MOUSE	Q9Z130	122	-1.18	0.044	ubiquitin
**30**	Protein disulfide-isomerase A3	PDIA3_MOUSE	P27773	234	1.09	0.045	mitochondrion
**31**	Aconitate hydratase, mitochondrial	ACON_MOUSE	Q99KI0	437	1.08	0.046	metabolic process
**32**	Complexin-1	CPLX1_MOUSE	P63040	59	-1.18	0.05	synaptic proteins

Table S2. Differentially expressed proteins between FUS/MB-treated and sham-treated 3×Tg-AD mice.

No	Protein Name	Accession No.	Gene name	Mascot Score	Ratio	p-Value	Classification
**1**	Synapsin-1	SYN1_MOUSE	O88935	139	1.18	0.038	synaptic proteins
**2**	Fructose-bisphosphate aldolase A	ALDOA_MOUSE	P05064	64	-1.22	0.036	mitochondrion
**3**	ATP synthase subunit d,mitochondrial	B1ASE2_MOUSE	B1ASE2	307	1.11	0.0098	mitochondrion
**4**	Tubulin beta-2A chain	TBB2A_MOUSE	Q7TMM9	373	1.1	0.011	microtubule process
**5**	Ubiquitin carboxyl-terminal hydrolase isozyme L1	UCHL1_MOUSE	Q9R0P9	222	1.13	0.017	ubiquitin
**6**	Neurofilament medium polypeptide	NFM_MOUSE	P08553	99	1.39	0.014	microtubule process
**7**	Growth factor receptor-bound protein 2	B1AT92_MOUSE	B1AT92	243	1.13	0.018	others
**8**	Triosephosphate isomerase	TPIS_MOUSE	P17751	265	1.09	0.019	glycolytic process
**9**	MAGUK p55 subfamily member 2	MPP2_MOUSE	Q9WV34	46	-1.22	0.022	synaptic proteins
**10**	Tubulin alpha-1A chain	TBA1A_MOUSE	P68369	108	1.09	0.023	microtubule process
**11**	Neuronal pentraxin-1	NPTX1_MOUSE	Q62443	134	-1.09	0.032	synaptic proteins
**12**	Transgelin-3	TAGL3_MOUSE	Q9R1Q8	95	1.11	0.032	synaptic proteins
**13**	Heat shock protein HSP 90-beta	E9Q3D6_MOUSE	E9Q3D6	42	-1.17	0.00097	mitochondrion
**14**	Dihydrolipoyllysine-residue succinyltransferase component of	ODO2_MOUSE	Q9D2G2	107	-1.08	0.016	mitochondrion
**15**	Alpha-enolase	ENOA_MOUSE	P17182	240	1.26	0.042	glycolytic process
**16**	Guanine nucleotide-binding protein G(o) subunit alpha	GNAO_MOUSE	P18872	135	1.29	0.042	others

**17**	14-3-3 protein epsilon	1433E_MOUSE	P62259	78	1.12	0.044	microtubule process
**18**	Phosphoglycerate mutase1	PGAM1_MOUSE	Q9DBJ1	295	1.21	0.046	glycolytic process
**19**	Dihydropyrimidinase-related protein 2	DPYL2_MOUSE	O08553	75	1.38	0.039	synaptic proteins
**20**	Thiomorpholine-carboxylate dehydrogenase	CRYM_MOUSE	O54983	353	1.15	0.049	mitochondrion
